# χ-SOM: emergent self-organizing maps for large chemical space visualization

**DOI:** 10.1186/s13321-026-01317-z

**Published:** 2026-10-18

**Authors:** Johannes Kaminski, Oliver Koch

**Affiliations:** Institute of Pharmaceutical and Medicinal Chemistry, Corrensstraße 48, 48149 Münster, Germany

**Keywords:** Emergent self-organizing maps, Dimensionality reduction, Clustering, Chemical space visualization, Chemography, Machine learning, Software

## Abstract

The exploration and visualization of chemical space using dimensionality reduction (DR) methods are fundamental to cheminformatics, as they enable the exploitation of the similar property principle. However, most methods, including t-SNE and UMAP, struggle to preserve valuable distance and density properties from ambient space in the latent space representation. The emergent self-organizing map (ESOM), an often-ignored extension of the self-organizing map (SOM) algorithm, demonstrates projection behavior that circumvents the disadvantages of these methods. As a suitable implementation of this algorithm for large cheminformatic datasets is not available, we present χ-SOM, the ChemInformatics (ChI)-SOM library, a high-performance framework for training emergent self-organizing maps on large molecular datasets and for interactively exploring the resulting projections. χ-SOM integrates an optimized implementation of the SOM algorithm with CPU and CUDA backends, an on-disk HDF5-based datastore with PyTorch DataLoader support for efficient parallel access to millions of fingerprints, and a graphical user interface tailored to chemical structure visualization and property overlays. We compared our implementation with t-SNE and UMAP on synthetic and chemical data benchmarks that probe density and inter‑cluster distance preservation. On the synthetic benchmarks, ESOM retained both properties where t‑SNE and UMAP did not. On the fingerprint-based equivalents, all three methods performed comparably. χ-SOM was also benchmarked against established SOM libraries across varying dataset sizes and fingerprint lengths, demonstrating competitive or superior performance. As a final proof-of-concept, we successfully trained an ESOM of the ChEMBL33 database (1.7 million compounds; 5.1 million neurons), to our knowledge the largest SOM reported to date, and show that local neighborhood relationships on the map align well with medicinal chemists’ intuition of chemical similarity. χ-SOM thus provides a practical, extensible tool for large scale exploratory analysis of chemical space.

**Supplementary Information:**

The online version contains supplementary material available at https://doi.org/10.1186/s13321-026-01317-z.

## Scientific contribution

Here we introduce χ-SOM, a high-performance framework for training emergent self-organizing maps (ESOMs) with a specific focus on cheminformatics, including a datastore and interactive GUI tailored to this domain. We benchmark ESOMs trained with our implementation against t-SNE, UMAP, and existing SOM libraries on synthetic and real-world datasets, showing that ESOM preserves density and inter‑cluster distance relationships on synthetic benchmarks where t‑SNE and UMAP do not, that all three methods perform comparably on the fingerprint-based equivalents, and that χ‑SOM offers improved scalability against current SOM implementations. Finally, we construct, to our knowledge, the largest ESOM of chemical space reported to date (ChEMBL33, 1.7 million compounds; 5.1 million neurons), and show that the resulting projections align closely with medicinal chemists’ intuitive notion of chemical similarity.

**Supplementary Information:**

The online version contains supplementary material available at https://doi.org/10.1186/s13321-026-01317-z.

## Introduction

The concept of “chemical space” can be understood in two distinct ways. In its broadest sense, it represents the combinatorial space encompassing all conceivable chemical entities with reasonable estimates exceeding 10^60^ molecules [1]. As this space is so vast and not yet resolved by currently available libraries [2, 3], the term lacks a clear definition. Instead, it is often used in a more constrained sense to denote the chemical diversity of a set of compounds. In these sets, each compound is characterized as a vector of properties with these vectors defining a high-dimensional, ambient space.

In medicinal chemistry, the investigation of this space for similarity relationships is of special interest. When used with care, structural similarity, encoded in vector representations, can lead to the discovery of better-suited or more active compounds when analyzed computationally [4]. This behavior is often referred to as the “similar property principle”.

Although close analogs in a dataset can be identified through clustering and the investigation of close matches alone, visualization of the wider relationships can be highly beneficial for understanding the problem at hand [5, 6]. This form of chemical space visualization was first termed “chemography” [7]. It is most often achieved by applying dimensionality reduction directly to the vector representation or to tables of pairwise distance relationships. For an overview of the different methods employed, the reader may consult reviews [8–10].

Beginning in the early to mid-1990s, self-organizing maps (SOMs) [11] were used extensively for this task, ranging from visualization of molecular surfaces [12] to library design [13], compound screening [14], and beyond. This method projects datapoints onto discrete positions on a two-dimensional grid of neurons, with the neuron at the corresponding position referred to as the best-matching unit (BMU). The BMUs are determined by distance to weight vectors associated with each neuron, which are learned during training. This training iteratively adjusts the weight of the neurons such that they act as prototypes or centroids of a given subset of the dataset, with neighboring neurons in the low-dimensional embedding space being similar to each other in the high-dimensional ambient space.

With the availability of newer dimensionality-reduction algorithms, such as t-SNE [15] and UMAP [16], the use of SOMs in cheminformatics has declined. This may be attributed to several factors: the absence of a clear statistical framework and related issues [17], and, likely in significant part, the computational complexity that made it infeasible to use this algorithm with chemical datasets that are ever-increasing in size.

The trend toward using other, more recent, algorithms for dimensionality reduction is not limited to computational chemistry, and the use of self-organizing maps has declined overall. As a result, implementations of the SOM algorithm are scarce, often limited in their features with respect to parallelization and analytical tools, and therefore of limited use for creating large maps for cheminformatic applications. In particular, exploring the chemical space often requires investigating the resulting map and the mapped datapoints. However, human comprehension of chemical structure is much more intuitive when depicted as a chemical graph than in one-line notation. This makes the interactive investigation of the resulting map highly desirable.

While the standard SOM may fall short of the projection quality achieved by prototype-free approaches such as UMAP and t-SNE, Ultsch et al. later introduced an extension of the SOM algorithm, called emergent SOMs (ESOM) [18]. They proposed maps with multiple neurons per datapoint, combined with the U-Matrix, now a staple of SOM visualization (see Fig. 1). This U-Matrix indicates the distance in the ambient space between a neuron and its next-neighbor neurons in embedding space and serves as background for the projection area. The positions of the BMU, together with the U-Matrix, enable interpretation of the resulting maps, which are very similar to positions on geographic surface maps. Regions of the map with consecutively low U-height indicate that entities mapped to these regions form a cluster in ambient space. Regions of large U-height indicate outliers or separation between clusters. Together with contour plots or color coding of the U-Matrix, this results in ‘valleys’ of similar entities and ‘ridges’ separating them. In our view, this extension substantially increases the interpretability of the map, as the U‑Matrix makes cluster boundaries and their relative separation directly readable, information that t‑SNE and UMAP embeddings do not convey. However, as this form of SOM significantly increases the number of neurons, it also exaggerates the already unfavorable computational demand.

**Fig. 1:**
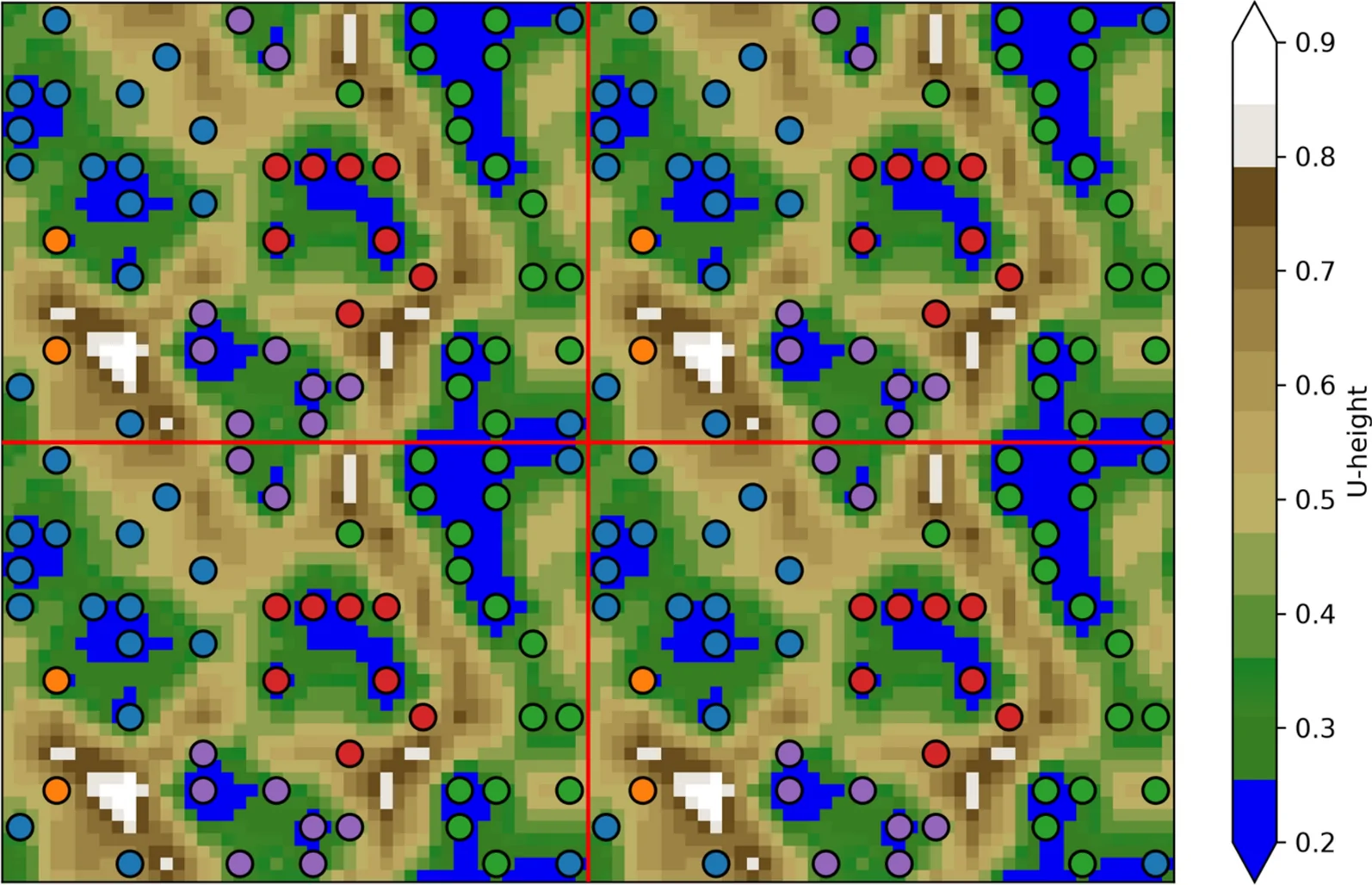
Example of a 2 × 2 tiled U-Matrix plot. Blue and green background colors for ‘valleys’, regions of small distance between neurons in ambient space, indicating clusters such as with the purple and yellow BMUs. Brown to white colors for ‘ridges’, regions of large distance between neurons in ambient space, indicating cluster boundaries. Marker colors show data class membership. Red line shows tile border. Tiling shows a continuous, toroidal, surface that the compounds are projected on

These requirements led us to investigate available implementations for cheminformatic applications. However, none met the criteria, prompting us to implement the algorithm described below. We analyze the capabilities and shortcomings of the different implementations, including performance benchmarks.

The original authors of both t-SNE and UMAP state that, while preserving topology well, both algorithms are known to distort absolute distances in ambient space between datapoints in the resulting embedding [15, 16]. These distortions can lead to misinterpretation and misuse of both methods in visual analytics [19]. To evaluate the sensitivity of ESOM to this phenomenon, we compare ESOM to t-SNE and UMAP on simple synthetic benchmark sets for dimensionality reduction previously established for both [20, 21]. To analyze the impact of this behavior in cheminformatic applications, we applied the same methods to constructed chemical datasets similar in topographic properties to the synthetic benchmarks. We conclude our work by applying the emergent version of the SOM algorithm to the ChEMBL33 [22] dataset, which contains 1.7 million compounds. This results in the training of a map comprising 5.1 million neurons, likely making it the largest SOM to date. We show that projections of this size still yield representations that align well with medicinal chemists’ understanding of chemical similarity.

## Materials and methods

### Self-organizing map

In self-organizing maps, the relationship between the high-dimensional input from ambient space and the low-dimensional projection is represented as an arrangement of neurons. All neurons n∈N are placed on an ordered lattice, most commonly a quad lattice with four neighbors or a hex lattice with six neighbors. The positions of the neurons ${p}_{n}$ remain consistent throughout training and are an important feature for determining neighborhood during the collaboration phase. Each neuron is represented as a vector of weights ${w}_{n}$ of the same length and data type as the input datapoints $v \, \in V$. After training, the weight vectors of each neuron should be close to a subset of datapoints in ambient space, with neighboring neurons in the embedding space presenting similar subsets of datapoints in ambient space [11].

#### Online algorithm

The original version of the algorithm is the online version. It comprises three phases, shown below. Importantly, each datapoint is presented individually, and the neuron weights are updated after each datapoint [11].

1. Competition:
  For a given datapoint, all the neurons on the lattice compete for which one is best in representing the datapoint using the minimum distance between the datapoint v and the weight vectors of all neurons (Eq. 1). The most widely used distance measures are the Euclidean distance (Eq. 2) and Manhattan distance (Eq. 3). Alternatively, similarity measures can be used and with the best-match and update equations (Eq. 1 and Eq. 6) adapted accordingly. Here, Cosine similarity is a common choice (Eq. 4). The selected neuron is called the best-matching unit.
  1 $${n}^{*}=arg\underset{n \in N}{min}\left(v, {w}_{n}\right)$$
  where ${n}^{*}$ is the best-matching neuron, v is the current datapoint and ${w}_{n}$ is the weight vector of a neuron
  2 $${d}_{x, y}=\sqrt[2]{{{\sum}_{i=1}^{I}\left({x}_{i}-{y}_{i}\right)}^{2}}$$
  3 $${d}_{x, y}= {\sum}_{i=1}^{I}{x}_{i}-{y}_{i}$$
  4 $${s}_{x, y}= \frac{{\sum}_{i=1}^{I}{x}_{i}{y}_{i}}{\sqrt[2]{{\sum}_{i=1}^{I}{x}_{i}}\sqrt[2]{{\sum}_{i=1}^{I}{y}_{i}}}$$
  where x,y is any pair of same length vectors.
2. Collaboration
  The degree of neighborhood in the embedding space is determined for all SOM neurons relative to the BMU. Commonly, a Gaussian-shaped distribution is used for (Eq. 5), based on either Euclidean (Eq. 2) or Manhattan (Eq. 3) distance for d. To achieve convergence of the map, the neighborhood size is continuously decreased during training at each timestep.
  5 $$\Lambda \left(n, {n}^{*}\right)=exp\left(-\frac{{d}_{n, {n}^{*}}}{2{\sigma}_{\Lambda }^{2}}\right)$$
  where ${d}_{n, {n}^{*}}$ is the distance in embedding space between any vector n and the best-matching neuron ${n}^{*}$, and ${\sigma}_{\Lambda }$ is the current size of the neighborhood.
3. Update:
  Finally, the weights of all neurons in the SOM are updated by some value Δw, depending on the current difference between the neuron and the datapoint at each vector position, the degree of neighborhood in embedding space and the learning rate (Eq. 6). The learning rate too is continuously decreasing over the course of the training to achieve convergence.
  6 $$\Delta {w}_{n}= \alpha \left(t\right){\Lambda}_{n,{n}^{*}, \sigma (t)}\left(v-{w}_{n}\right), \forall n \in N$$
  where α(t) is the learning rate at the current time step t, $\sigma \left(t\right)$ is the neighborhood size at the current time step t

#### Batch algorithm

The batch algorithm is a revised version that falls outside the theoretical framework proving convergence of the original algorithm, but it has also been shown to converge in practice. Unlike the original algorithm, competition and collaboration are performed for all datapoints without intermediate updates. Instead, the new weight vectors of the neurons are averages over all datapoints, weighted by the degree of neighborhood at each neuron (Eq. 7). This architecture increases parallelizability and reduces both the number of operations and the number of memory accesses, significantly improving computational performance [23]. Some drawbacks of this version of the algorithm, particularly with the ESOM variant, can be resolved by applying the update after a 1/k-sized subset of the original dataset, termed *the k-batch* algorithm [24].

7 $${w}_{n}\left(t+1\right)= \frac{{\sum}_{v\in V}\Lambda \left(n, {n}^{*}\left(v\right)\right)v}{{\sum}_{v\in V}\Lambda \left(n, {n}^{*}\left(v\right)\right)}$$

#### U-distance

Due to the nature of the ESOM algorithm, even under perfect topology preservation it is common that a neuron is separated by large distance in ambient space to its direct neighbor in one direction and small distance in ambient space to neurons multiple grid positions over in another direction. Even though this property is communicated to the user by the U-Matrix representation, this can lead to distortions when applying forward projection error measures to ESOM projections, as distance in embedding space is most commonly measured by p-norm distances. To capture the distance differences communicated via the U-Matrix in the projection error, Ultsch et al. proposed a new measure of forward projection error, including a full discussion on the problem, the Minimal U-Ranking Measure [25].

While the full measure is reasonable for comparing ESOM under different hyperparameter configurations, it is inapplicable to other methods of DR and not useful for direct comparison. However, it also includes a novel distance measure (Eq. 8) named *U-distance*. We propose that this distance measure can be used in other rank- and correlation-based projection error measures as the embedding space distance measure for the ESOM. This would allow for fair comparison of ESOM to other methods of DR, preserving distance differences captured by the U-Matrix.

${PATH}_{i,j}$(Eq. 8) is the set of arbitrary paths between neurons i and j. ${N}^{M}\left({i}_{k}\right)$ is the set of next neighbor neurons to neuron ${i}_{k}$

8 $$\begin{aligned}&PATH_{i, j}=\{(n_1,...,n_p): \mathbb{N}{\backslash}\{0, 1\},\\&n_1=i,n_p=j,n_{k+1}\in N^M(n_k)\}\end{aligned}$$

$pathdistance\left(.\right)$ is the length of such a path, based on the ambient distance d of the neurons.

9 $$pathdistance\left({n}_{1}, \dots ,{n}_{p}\right)= \sum_{k}^{p-1}d\left({w}_{{n}_{k}}, {w}_{{n}_{k+1}}\right)$$

$udistance\left(i, j\right)$ is the minimum path distance between neurons i and j.

10 $$u\mathit{distance}\left(i,j\right)=\underset{q\in {\mathit{PATH}}_{i,j}}{\mathrm{min}}pathdistance(q)$$

### t-distributed stochastic neighbor embedding

T-distributed Stochastic Neighbor Embedding (t-SNE) is a prototype-free dimensionality reduction method that aims to arrange the positions of datapoints in a low-dimensional embedding such that their distance distributions approximate those in the original, high-dimensional ambient space [15]. For this, a Gaussian is placed over each datapoint in the ambient space. The distances of all other datapoints to this centroid are commonly measured by Euclidean distance (Eq. 2). The width of the distributions at each datapoint is chosen such that the sum of likelihoods of all datapoints under the distribution is the same for each distribution. The *perplexity* hyperparameter determines the extent of this scaling.

In the embedding, the positions of the datapoints are initialized either randomly or set to a predetermined initial position, often an embedding generated from principal component analysis (PCA) [26, 27]. Again, distributions are fitted in the embedding space, this time using a Student t-distribution. Gradient descent optimization is performed to create clusters in the low-dimensional embedding, that are similar to the clusters in the ambient space. Iteratively, the positions of the datapoints in the projection are updated according to a cost function based on the Kullback–Leibler divergence between each datapoint’s distributions in the ambient and embedding spaces.

### Uniform manifold approximation and projection

Uniform Manifold Approximation and Projection (UMAP) is a prototype-free dimensionality reduction method that arranges datapoints in the low-dimensional embedding such that their distances resemble those in a neighborhood graph built from the high-dimensional ambient positions [16]. First, similarity scores are assigned from each point to all other points. The similarity scores of the first *n-1* (*n* = *n_neighbors*) neighbors sum to a value that is also determined by *n*. From these pairwise similarity scores, a neighborhood graph is constructed. From the edges of the neighborhood graph, the positions in the projection are initialized, often via spectral embedding, PCA or at random. Low-dimensional similarity scores from each datapoint to all other datapoints are now assigned using a scaled t-distribution centered at each low-dimensional position. The positions are then iteratively optimized using stochastic gradient descent. Here, the cost function rewards low-dimensional similarity scores between a randomly selected datapoint and a second random datapoint from the set of n-neighbors in ambient space, while penalizing low-dimensional similarity scores between the datapoint and a third random datapoint not in the set of neighbors.

### ChEMBL33 dataset

ChEMBL33 is a compiled library of activity data and contains information on approximately 2.2 million compounds, 20 million activity measurements, and 15,000 targets. The information has been manually compiled from approximately 85,000 publications. For our purposes, the raw dataset was prepared using the RDKit [28] to remove salts as well as tautomeric and stereochemical duplicates, resulting in approximately 1.7 million distinct compounds.

### Evaluation of the self-organizing map implementations

Available libraries implementing the SOM algorithm were compiled from the literature and GitHub and are listed in Table A1. All software libraries that offer either a CPU- or GPU-parallel implementation of the algorithm were benchmarked [29–32]. Somoclu [33] was excluded as it is incompatible with new GPUs due to use of a deprecated CUDA version and does not receive further updates. If available, both the batch and online variants were assessed, along with the CPU and GPU implementations. If available, Euclidean and Cosine similarity metrics were investigated.

To assess the performance of our χ-SOM library and compare it to other libraries, test sets of sizes 100, 1000, 10,000 and 100,000 compounds were created by randomly sampling from the prepared ChEMBL33 data three times each. For each dataset, fingerprints of sizes 512, 1024, and 2048 were generated using the RDKit Morgan fingerprint implementation [28]. The number of neurons per map was set to three times the number of datapoints in accordance with the ESOM heuristics proposed by Ultsch et al. [18]. Single precision floating point representations were used for each weight. The efficiency of each implementation was benchmarked by comparing both memory usage and compute time per epoch. Each datapoint was presented five times (i.e., five epochs). (Note: Using ‘epoch’ in this way for both online and batch implementations diverges from the literature, where ‘epochs’ refers to a single pass over the whole dataset only for batch implementations. For online implementations, ‘epoch’ or ‘k’ refers to the presentation of a singular datapoint and subsequent update of the whole codebook.) For compute time measurement, the results across all epochs for each set with the same fingerprint size and number of datapoints were pooled, yielding 15 samples and improving accuracy. To avoid overhead in the runtime measurement, memory consumption was measured in an independent experiment with the same setup. Memory usage of the ESOM computation was polled at intervals by a secondary process until the training of the ESOM finished, or memory usage had stabilized.

All tests were performed on a high-performance computing system running Rocky Linux 9. For CPU-accelerated calculations, each task was allocated 24 cores of an AMD EPYC 7713 and 128 GB of DDR4 memory. For GPU-accelerated calculations, 2 cores, 128 GB of DDR4 memory, and a NVIDIA GeForce RTX 4090 were allocated.

### Benchmark of ESOM on common dimensionality reduction difficulties

As demonstrated by Wattenberg et al. for t-SNE [20] and Coenen and Pearce for UMAP [21], certain features may be missed by these methods of DR. To analyze whether ESOM exhibits similar shortcomings, we re-constructed the density and inter-cluster distance synthetic datasets. Additionally, datasets that translate density and distance properties of the synthetic datasets into cheminformatic problem sets were constructed using small molecule fingerprints. These were then used to gauge the implication of these properties for real world cheminformatic use.

Projections were performed using the scikit-learn [34] t-SNE and umap-learn [35] UMAP implementations. *perplexity* was set to values of 5, 15, 30, 50, and 100, with the *n_neighbors* hyperparameter for UMAP set to the same values, respectively. Even though not mathematically equivalent, both values govern a shift of focus from local to global topographic relationships when increased. We therefore vary them over the same numerical range and compare them on that basis, rather than treating them as interchangeable. For both algorithms these hyperparameters are considered to be the most important to optimize to achieve good projection performance, as described in both original literature and library documentation.

For t-SNE, we further considered *early_exaggeration* with values of 6, 12, and 24, with 12 being the default. Both *random* and *pca* initialization were evaluated. *learning_rate* remained unchanged as for datasets of this size heuristics propose values significantly lower than the authors’ minimum recommended value.

For UMAP further evaluated were *min_dist* values 0.1, 0.25, 0.5, 0.8 and 0.99. This hyperparameter controls whether embeddings appear more clustered and clumped or are dispersed more evenly across the projection surface. As initialization methods *spectral*, *random* and *pca* were analyzed.

For ESOM further evaluated were *learning_rate* with values 0.2, 0.4, 0.6, and *learning_rate_decay*, as well as *neighborhood_decay*, both with values of “linear” and “exponential”. A previous analysis by Ultsch et al. on datasets very similar to the ones used here found that a non-toroidal boundary condition leads to significant edge artifacts and should be avoided. The same analysis also found that a rectangular map shape should be preferred over a square one [25]. Map size was kept at three times the number of datapoints, as this is the defining feature of emergent SOM over standard SOM.

All algorithms were run using the Euclidean distance measure. For each hyperparameter configuration three replicates with random seed 42, 8,529,492 and 9,240,666 were run.

#### Synthetic datasets

Synthetic datasets (Fig. 5, first column) for the proposed experiments:

- Two clusters of the same number of datapoints drawn from two Gaussian distributions with different standard deviations. This aims to demonstrate the method’s responses across different intra-cluster densities.
- Three clusters drawn from Gaussian distributions with the same standard deviations, but with the means of distributions one and two closer than the means between two and three. This aims to demonstrate the method’s responses across different inter-cluster distances.

#### Chemical compound datasets

Molecular datasets were constructed from the ChEMBL33 library. First, fingerprints were generated using the RDKit *Morgan fingerprint* implementation with a *radius* of 2 and *fpSize* of 1024. The fingerprints were then clustered using the BitBIRCH [36] algorithm, supplied by the bb-lean library [37]. Default parameters were used except for *threshold*, which was set to the recommended 0.35 for Morgan fingerprints. To construct a chemical dataset similar to synthetic dataset *A,* with two clusters of different density, the constructed BitBIRCH tree was refined using a *tolerance* of 0.02. Only clusters with 100 < number of compounds < 150 were considered. We defined “density” as the ratio of average Tanimoto similarity of compounds within an identified cluster over the number of compounds in the cluster. This average Tanimoto similarity is a core feature of the BitBIRCH algorithm and termed *iSIM*. From the remaining clusters, those with the lowest and highest density were selected to make up the dataset.

To construct a chemical dataset similar to synthetic dataset *b*, with three clusters separated by different inter-cluster distances, the original constructed BitBIRCH tree was refined using a *tolerance* of 0. Only clusters with 50 < number of compounds < 200 were considered. For the remaining clusters, the pairwise Jaccard distance between all cluster centroids was computed using SciPy’s *pdist* [38]. Sets of three clusters were then formed, where two clusters had a pairwise Jaccard distance for cluster centroids between 0.45 and 0.55. The third cluster was chosen such that its centroid Tanimoto similarity to both other clusters’ centroids was larger than 0.95. From all possible three-cluster combinations, three were chosen at random. As a baseline, PCA plots were generated for each dataset using scikit-learn. Embeddings were then generated with all three DR methods at the same hyperparameter settings stated in the synthetic benchmark dataset evaluation.

To gauge comparative embedding performance, Shepard diagrams [39] were created plotting Tanimoto similarity on one axis against the embedding distance measure. This distance measure was Euclidean distance for both t-SNE and UMAP and U-distance for ESOM. Projection performance was further analyzed by comparing Spearman correlation coefficients.

Additionally, k-Nearest-Neighbor (k-NN) retention/preservation [40] analysis was performed on the full dataset, measuring the average fraction of *k* neighbors to a datapoint in ambient space, that is also in the set of the *k* closet datapoints in embedding space. This can indicate how well local relationships are preserved in the embedding. While for t-SNE and UMAP the ranking of datapoints by proximity is most often unambiguous, occupation of the same neuron by multiple datapoints is not rare. The grid-based nature of the embedding can also lead to the same distance for multiple neurons. This may result in ambiguous set of nearest neighbors in embeddings space. To break such ties in an unambiguous way, if the last k-neighbor in embedding space is shared among multiple datapoints, the relative number of them being in the set of k-neighbors in ambient space is used. For t-SNE and UMAP Euclidean distance was used as the embedding space distance measure. For ESOM u-distance was used.

### Applicability of the ESOM algorithm to large cheminformatic datasets

To showcase the capabilities of χ-SOM and assess whether the ESOM algorithm can generate a meaningful embedding of an extensive chemical dataset, we trained an ESOM on the large dataset of Morgan count fingerprints. Fingerprints with a length of 1024 and a *radius* of 2 were generated from the previously prepared ChEMBL33 dataset. The ESOM was configured according to the heuristics previously established by Ultsch et al. [25], including a rectangular map shape with an edge ratio of approximately 1:1.5, three times as many neurons as datapoints, and a 2D toroidal projection area (periodic boundary conditions). This corresponds to a map of 1887 by 2829 neurons leading to a overall number of 5,338,323 neurons and, in combination with the fingerprint length, of 5,466,442,753 weights. Single precision floating point representations were used for each weight. Learning rate decay was set to a linear cooling strategy, with an initial rate of 0.8. Neighborhood size decay was set to an exponential cooling strategy with an initial radius of 943 neurons. Cosine similarity was used during training for a fixed duration of 30 epochs instead of a convergence criterion, while Euclidean distance was used for BMU calculation for the final stage, as it tends to give finer local resolution. A batch size of 1000 was used. The random number generator used within χ-SOM was set to a seed of 42.

The trained map was evaluated using drug series from ChEMBL33. First, we selected the groups of gyrase-inhibiting and beta-lactam antibiotics since most compounds in these series share overall structural similarities, but contain subclusters, such as the penam, penem, cephem, and monobactam series for antibiotics from the beta-lactam series. Analysis was conducted manually by examining the local relationships among the corresponding compounds in the embedding projection, including those from ChEMBL33 projected into their direct neighborhood.

Shepard diagram/Spearmen correlation and k-NN retention analysis of the ESOM was performed analogous to the previous experiment. However, as calculation of u-distance for all datapoint combinations is infeasible, sampling was used. Due to the size of the dataset, it becomes increasingly unlikely to sample next neighbors with smaller samples sizes. To still be able to both include local and global relationships in the Shepard diagram at 4000 samples, half of the samples were collected randomly from all datapoints and half from within same clusters. Clustering of the dataset was performed using bb-lean [37] using the same setting from the previous experiment for the construction of the distance datasets. Furthermore, all pairwise Tanimoto similarity relationships between compounds were characterized.

This experiment was performed on the same hardware described above.

## Results and discussion

### A new implementation of the SOM algorithm for ESOM in cheminformatics

#### χ-SOM

The χ-SOM, ChemInformatics (ChI)-SOM library (Fig. 2) consists of three main components, each providing specific capabilities for visualizing large chemical libraries. This split is deliberate. The computational power required to train very large (E)SOMs is often unavailable on local workstations, so training should be performed on compute clusters with headless environments. This way the *Som* object and *core* modules, as the compute-only part of χ-SOM, can be installed without the need for GUI libraries on these headless systems.

**Fig. 2:**
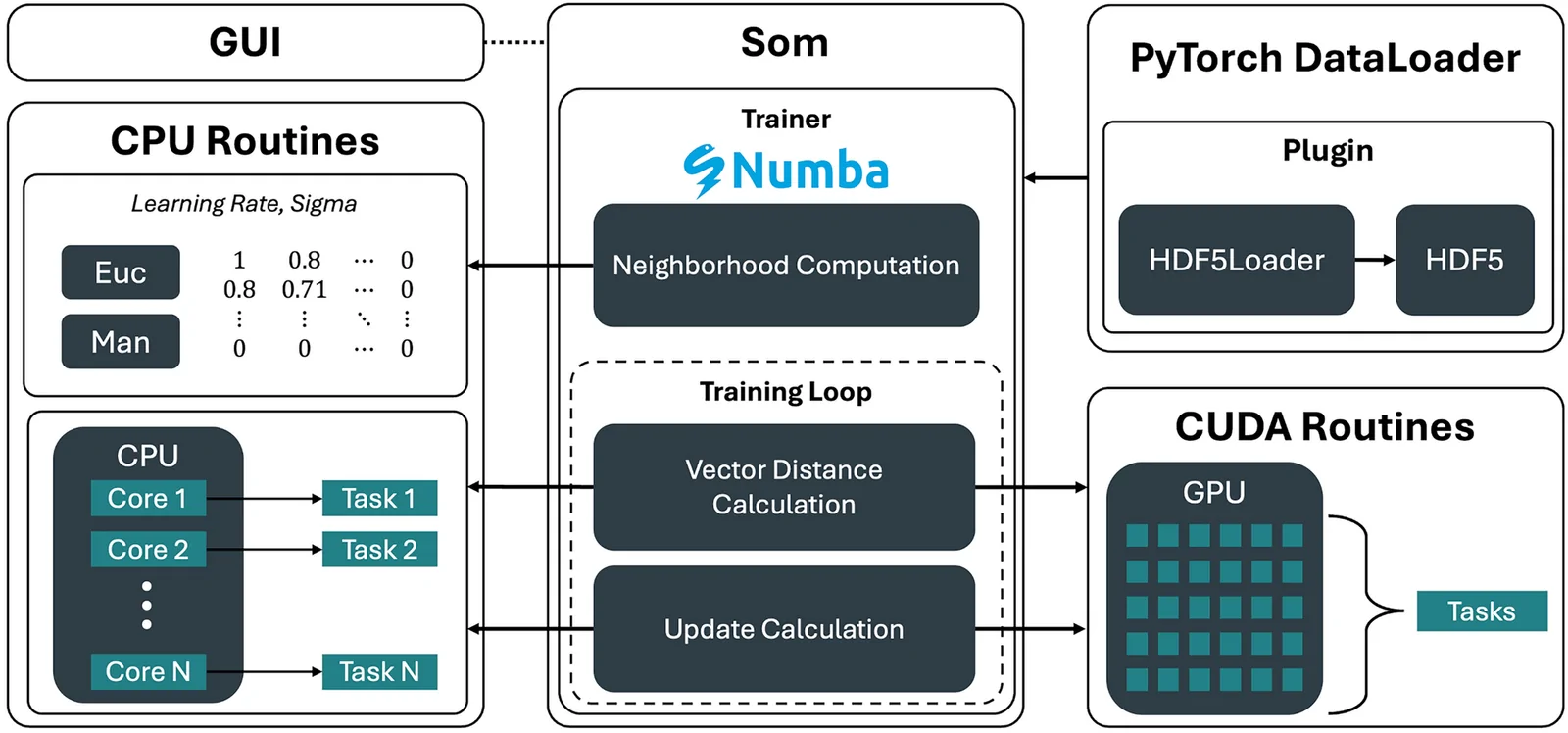
Overview of the χ-SOM architecture

To not put additional strain on RAM requirements for large ESOM calculation, χ-SOM provides an on-disk HDF5 based storage backend that can be used with PyTorch’s [41] DataLoader for pre-fetched, random access without read overhead. All other analyzed libraries only support preloading of all data into RAM.

The *gui* module enables the interactive visualization and analysis of chemical representations projected onto large (E)SOM maps. This feature is unique among all analyzed SOM implementations, which at most support simple BMU/U-Matrix plots (Table A1). Currently, Databionic ESOM TOOLS [42], developed in 2006, is still the de facto standard for analyzing ESOM. While results of other implementations can be translated for processing with this tool, due to its age it is incapable of handling maps with the desired millions of datapoints. Additionally, while investigation of clustering and class membership is rich, it does not support visualization of chemical features, desirable for cheminformatic analysis.

To position this work relative to the existing ESOM literature, we distinguish three levels of contribution. The ESOM concept, the U‑Matrix, the density and topology interpretations it enables, and the map-sizing heuristics are established results due to Ultsch and co-workers [25], and we adopt them unchanged. Our algorithmic contribution is twofold: the precomputed neighborhood/learning-rate lookup described below, which to our knowledge no other implementation employs. Furthermore, the availability of U-distance as the embedding-space metric in rank- and correlation-based projection-error measures, which, for small ESOM, makes ESOM directly comparable with other dimensionality-reduction methods. The remainder, the CPU and CUDA backends, the on-disk HDF5 datastore, and the cheminformatics GUI, is an engineering contribution that brings ESOM within reach at a scale previously unattainable.

##### Core

In combination with the main SOM object, the χ-SOM *core* library provides an easy-to-use interface for training the self-organizing map. When initializing the *Som* object, the user can specify hyperparameters that remain constant throughout training. These include the neuron grid size, the distance measure used in the ambient space (Manhattan, Euclidean, Cosine, Dot-product), the distance measure used in embedding space (Manhattan, Euclidean, Manhattan on a toroidal surface, Euclidean on a toroidal surface), and the shape of the neighborhood kernel (Gaussian, Mexican hat, cone).

In addition to these hyperparameters, χ-SOM supports two accelerators (CPU and CUDA-enabled GPUs) with different flags to improve performance at the cost of numerical accuracy. While most of the core algorithm is a reimplementation of the core SOM algorithm in the numba [43]/numba-cuda-mlir [44] framework, both CPU and CUDA implementations include an algorithmic improvement in calculating the update to the neurons. In all analyzed implementations, the distance of each neuron on the map to the BMU, and the subsequent scaling of the update to the neuron by the neighborhood value and the learning rate, are calculated at each iteration. This accounts for significant compute overhead. Instead, this can be unified to a single value and precomputed for each epoch. The values are stored in an array with the dimensions of the neuron grid, with the BMU values at [0,0]. Utilizing this, the calculation of the update to the neurons after each training step reduces to the absolute difference in grid position and the lookup of the corresponding value in the matrix. Subsequently, the difference between the weight vector and the data vector can be multiplied by this value as a single factor and added to the neuron’s weight vector. These last two operations are highly optimized in the microcode of modern hardware, often fused into a single multiply–add operation, making this operation very efficient. By analyzing the code of previous implementations, we established that other libraries have not utilized this improvement so far.

Similar to scikit-learn syntax, the training loop is fused into a single method *Som.train()*. Given the number of training epochs, the batch size, and the starting and decay conditions for *alpha* (learning rate) and *sigma* (initial neighborhood size), this allows the SOM to be trained in a single step. Additionally, finer-grained control over training hyperparameters is available through *Som.train_batch()*. While requiring manual handling of epochs, data shuffling, *alpha* and *sigma* decay, this finer control enables more complex training strategies such as pretraining with a reduced dataset, adding samples after the original training, and continuing a training run after an abort. To calculate the current learning rate *alpha* and neighborhood radius *sigma*, convenience functions are provided in *chisom.tools* that use linear and exponential cooling strategies as the base. A helper function to calculate the appropriate map dimension as a multiple of the dataset size is also available.

##### Dataloader

χ-SOM provides a *ChemDataset* plugin in the *io* module that enables access to an HDF5 storage backend via PyTables [45]. This format hierarchically organizes tabular data while allowing quick random access to individual entries within the file. At the top level, groups allow for subdivision of the dataset. This is useful for datasets with distinct splits, such as training and test compounds, and is necessary when a single set exceeds roughly 4.6 billion entries. At a second level, information on different properties is stored in distinct arrays, with individual entries connected by a common key. From these properties, χ-SOM subsequently uses ‘fingerprints’ to train the SOM, while ‘molecule’ stores structural information for later display by the GUI. Any number of additional properties can be stored and marked as either categorical or continuous for later display in the GUI as overlays on BMUs.

Since constructing such structured datasets by hand is tedious and error-prone, χ-SOM provides a script to generate the file for on-disk storage. A *DataloaderFingerprintGeneratorFactory* object must be passed to this script, which supplies distinct generators to each process of the storage generation routine. *rdStyleFactory* serves as an option for most conversions of text-based molecular representations, such as SMILES or InChI stored in CSV files, to fingerprints and structures. It requires both an RDKit *MolGenerator* to convert the input columns’ text format and an *rdFingerprintGenerator* to generate the fingerprint representation. Keyword arguments to the *rdFingerprintGenerator* can be passed as a dictionary. Furthermore, χ-SOM also provides a *CSVStyleFactory* that selects consecutive columns from a CSV row and stores them as that entry’s fingerprint.

##### GUI

χ-SOM provides the necessary tools to investigate large chemical datasets (Fig. 3). Its main view shows the U-Matrix of the ESOM with the BMUs projected onto it. This map can be panned and zoomed using the mouse, and BMUs can be selected using a lasso tool. Hovering over BMUs generates a popup window, showing structure and stored properties of the datapoints/compound associated with this BMU.

**Fig. 3:**
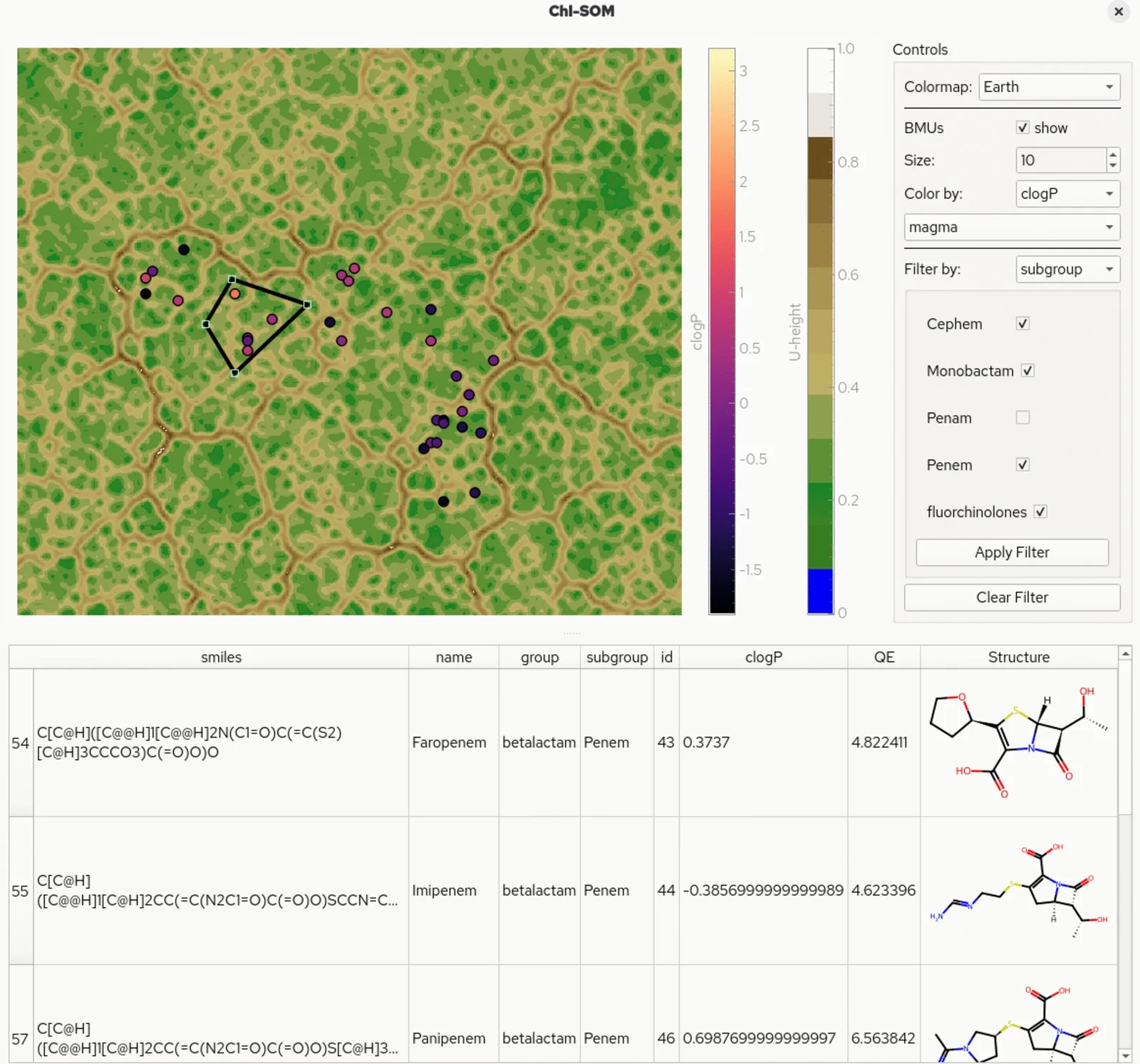
Overview of the χ-SOM graphical user interface

Selected entries are then displayed in a table view underneath the main view, showing any further information on the datapoint corresponding to the chosen BMUs, as well as the structure of the molecule. From the table view, entries can be selected, copied or pasted, or the entire table content can be exported.

The visualization of both the U-Matrix and the BMUs can be customized. Different color maps are available for the U-Matrix, and other information can be overlaid on the BMUs, with coloring available for both discrete properties, such as group membership, and continuous properties using gradient color maps. Additionally, datapoints and their corresponding BMUs can be filtered out and hidden from view, based on property ranges.

χ-SOM implements the user interface using the PySide6 framework for Qt [46] with the map area rendered using PyQtGraph [47].

#### Performance comparison with other libraries

From the runtime comparisons (Fig. A1), it is evident that all implementations exhibit a quadratic increase in runtime with increasing datapoint count. This is expected under the chosen constraint of ESOM with three times as many neurons as datapoints, resulting in O(n^2^) complexity. Using the heuristic proposed by Kohonen for the classic SOM approach instead, of one additional neuron every 50 datapoints [23], would lead to the same complexity, albeit at slower scaling. As the number of neurons however can be chosen freely, keeping it constant while increasing the number of training datapoints would only yield O(n) complexity. However, Ultsch has noted that choosing too small a number of neurons for the datapoint makes SOM behave more like k-means clustering [48]. Conversely, increasing vector length only yields a linear increase in the number of calculations and thus in runtime. The only library for which this is not true is sparse-som. sparse-som uses a sparse vector format, performing calculations only on the non-zero positions of a vector and omitting many calculations on vectors with many zero positions, such as fingerprints. Given the nature of Extended-Connectivity Fingerprints, the total number of set bits in a vector does not scale linearly with fingerprint length. Disregarding the relatively rare folding collisions that occur during halving of the fingerprint length, the number of set bits within the fingerprint remains similar when increasing fingerprint length [49]. This explains the minor increase in runtime per epoch when increasing the size of the fingerprints. Similar observations can be made for the memory usage (Fig. A2). All implementations exhibit quadratic memory scaling, due to the number of neurons increasing quadratically. sparse-som exhibits far lower memory requirements at the same scaling, due to non-zero elements stored as position/value pairs, with zero elements not consuming additional memory.

Comparing the most efficient implementations across all libraries (Fig. 4), it is evident that both the SimpSOM CPU batch and SuSi online implementations perform worse than our χ-SOM GPU online implementation. IntraSOM batch performs nearly on par. However, SimpSOM, SuSi, and IntraSOM encountered out-of-memory errors when processing datasets with more than 10,000 datapoints. Investigating the implementations, we established that this is due to the creation of pairwise distance matrices for vector-to-neuron distances during computation. As these also scale quadratically with the number of datapoints in the ESOM algorithm, this leads to the rapid increase in required memory shown in the second plot.

**Fig. 4:**
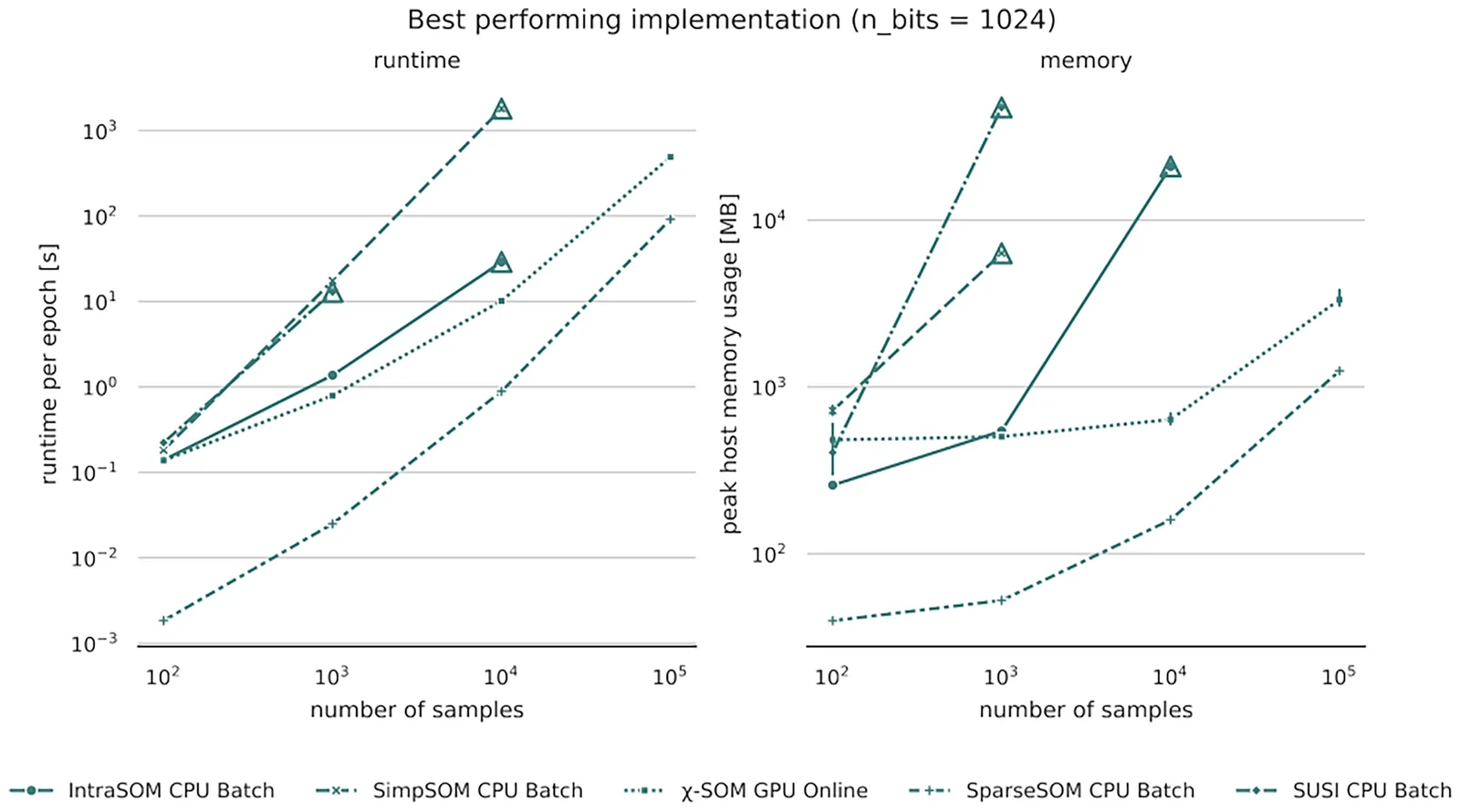
Runtime, in seconds per epoch, and memory usage comparison of the best-performing implementations of different libraries at a fingerprint length of 1024. Triangles mark the last measurable configuration. In this case next larger number of samples exceeded runtime or memory constraints. Absolute error bars are shown, although differences were often so negligible that they become invisible

The sparse-som batch implementation outperforms our χ-SOM GPU implementation by approximately one order of magnitude across all Morgan fingerprint sizes. However, although many structural fingerprints are quite sparse, this is not true for all cheminformatic fingerprints, such as interaction fingerprints. Performing computation on dense vectors with sparse-som, instead yields a linear increase in the number of computations (Fig. A3). Compared with other implementations, this results in worse runtimes, likely due to more inefficient memory access patterns. Furthermore, sparse-som only supports the Euclidean distance measure.

### Benchmark of ESOM on common dimensionality reduction pitfalls

#### Synthetic datasets

As previously described in the publications by Wattenberg et al. [20] and Coenen and Pearce [21] and theorized in the original publications on t-SNE [15] and UMAP [16] both methods struggle to preserve the density and inter-cluster distance relationships in the synthetic datasets at default hyperparameters (Fig. 5). Density properties of clusters are not reflected in the low-dimensional embedding at common values of *perplexity* (t-SNE) and *n_neighbors* (UMAP) (Fig. 5a). This extends to all combinations of hyperparameters analyzed (Fig. A8a/A9a, Supporting Information B).

**Fig. 5:**
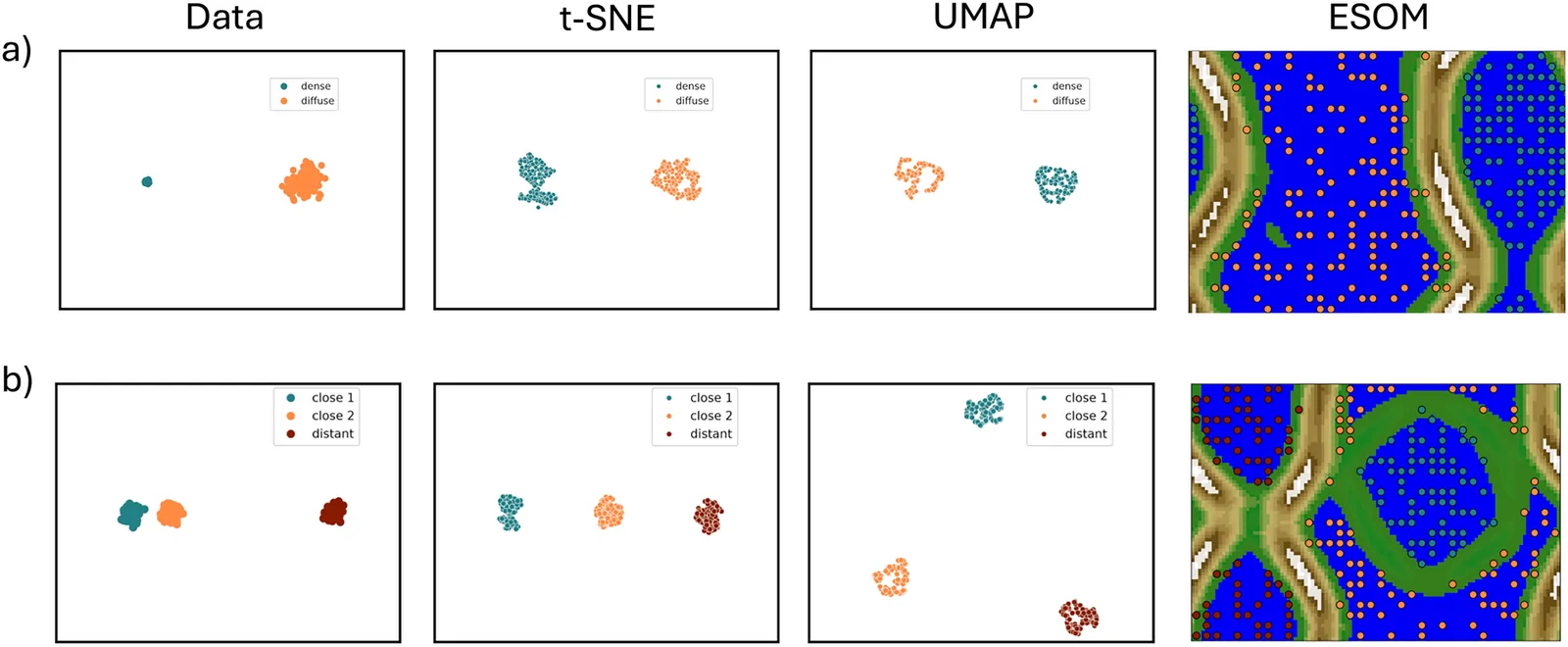
Example datasets that both t-SNE and UMAP fail to project while preserving dataset-specific features. a) Two clusters with different densities. b) Three clusters with different inter-cluster distances. All plots generated with default hyperparameter settings for each method and *seed*=42

Conversely, the ESOM architecture can capture these properties, as the basins corresponding to the denser cluster occupy less space in the resulting projection. Furthermore, BMUs for datapoints in the sparser cluster are farther apart in the projection. While this property may not be necessary for projections aimed at identifying cluster relationships, we argue that it is a property worth preserving, particularly in exploratory data analysis. In the concrete case of cheminformatics, less dense, i.e., sparsely populated, chemical space may indicate structural space worth further investigation, as it may contain new chemical entities, patentable space, or similar [50].

A similar observation can be made regarding both methods’ ability to preserve inter-cluster distance relationships (Fig. 5b). Increasing the *perplexity* or *n_neighbors* hyperparameter to roughly three time, or seven time respectively, the default value, both methods capture these properties in their embeddings (Fig. A8b/A9b). However, this is highly dependent on the value of the hyperparameter relative to the number of datapoints. As shown in Fig. A10, this information disappears as the number of datapoints increases. Variation in the other hyperparameters shows little influence on the resulting embedding for t-SNE. For UMAP, variation in both the *min_dist* and the chosen initial embedding (*pca* vs. *spectral* vs. *random*) influences the embedding slightly; however, the *n_neighbors* hyperparameter heavily dominates the resulting plot (Supporting Information B).

The ESOM projection shows a different behavior. All datapoints of the leftmost cluster in Fig. 5b, and thus furthest removed from the rightmost cluster, are embedded within the part of the map that corresponds to the center cluster. Given the toroidal nature of the projection area, these two are thus projected as far apart as possible. Cluster differentiation is again achieved by the U-Matrix, which shows low U-height for all three clusters, with ridges separating them. However, the ridge between the two left clusters has a lower U-height, corresponding to the shorter distance in ambient space. Furthermore, this property is independent of dataset size, seen in Fig. A10. This behavior is further stable across a wide spectrum of hyperparameter combinations (Supporting Information B). Other hyperparameters, such as the learning rate and the cooling strategies for both the learning rate and the neighborhood radius, in agreement with literature [23], only exhibit negligible effects.

In our opinion, this yields a more reliable representation of the ambient space, as it induces a sense of directionality and distance to movement within the projection. In contrast, the t-SNE and UMAP projections cannot establish this directionality.

#### Chemical compound datasets

Using the generated chemical compound datasets, all three algorithms exhibit the same general behavior observed with the synthetic dataset when embedding clusters with differing density (Fig. 6a). For both UMAP and t-SNE, the difference in density between the clusters is heavily distorted in the embedding, making both clusters appear roughly equally spaced. This behavior is stable across a wide range of hyperparameter combinations for both methods (Fig. A11a/A12a). However, smaller *n_neighbors* values for UMAP seem to perform marginally better (Fig. A12a). ESOM again captures cluster density differences by allocating more neurons to the less dense cluster and vice versa. Additionally, ESOM tends not to crowd the projection as seen for UMAP, and to a lesser degree t-SNE, and instead allows for more spacing in between the BMUs of the datapoints. This behavior is stable across a wide range of hyperparameter configurations (Supporting Information B) for the ESOM and for UMAP only decreases at high *min_dist* values of 0.8 (Fig. A13). The original authors already stated that this increase in *min_dist* tends to hide finer features in the projection [16].

**Fig. 6:**
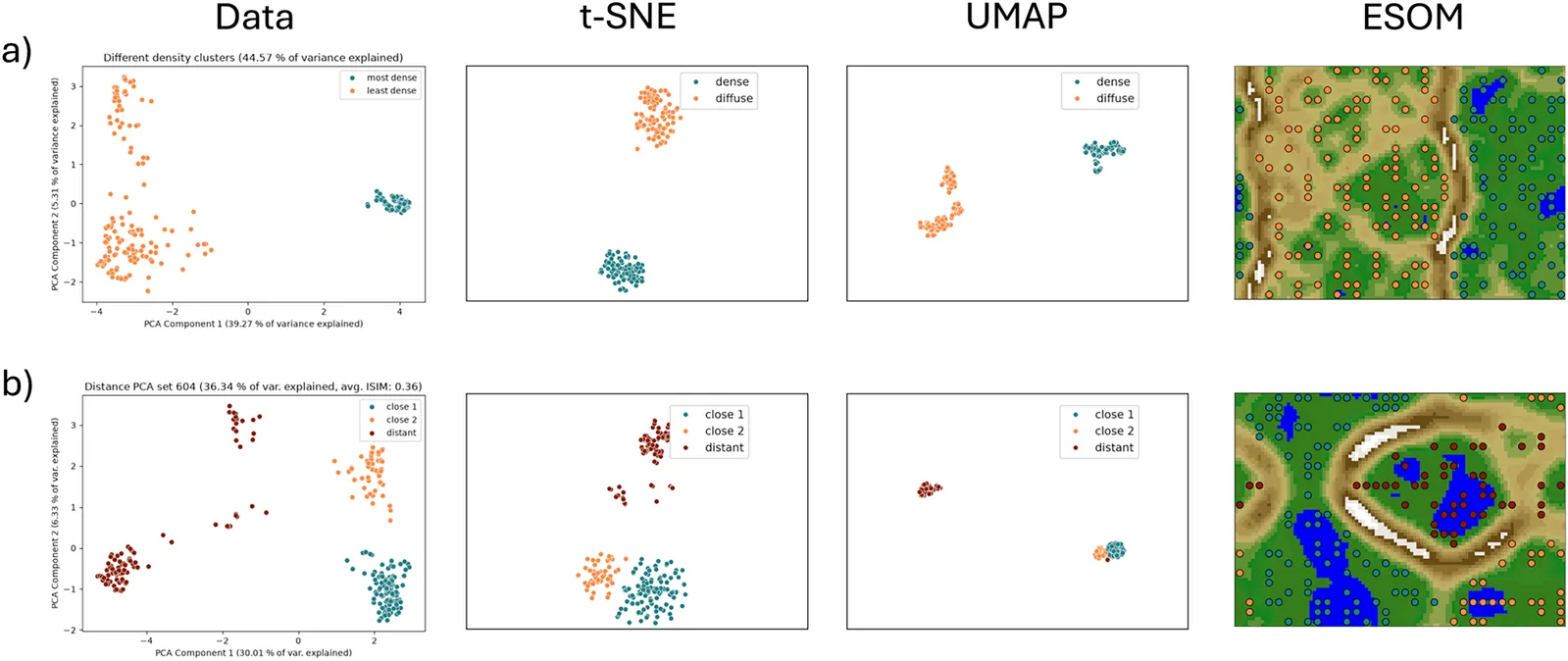
Projections of constructed compound datasets that mimic the synthetic datasets. The first column shows a PCA plot of the fingerprint data. **a** Two clusters with different densities. Resulting plots of run with best performance measured by k-NN retention shown for each method. t-SNE hyperparameter: early_exaggeration = 24, init = pca, perplexity = 50, seed = 42. UMAP hyperparameter: n_neighbors = 15, min_dist = 0.1, init = pca, seed = 42. ESOM hyperparameter: learning_rate = 0.4, lr_decay = linear, neighborhood_decay = exponential. **b** Three clusters with different inter-cluster distance. Resulting plots of run with best performance measure by pearson correlation shown for each method. t-SNE hyperparameter: early_exagerration = 12, init = pca, perplexity = 100. UMAP hyperparameter: n_neighbors = 100, min_dist = 0.1, init = pca, seed = 42. ESOM hyperparameter: learning_rate = 0.2, lr_decay = exponential, neighborhood_decay = linear

A different picture emerges for the datasets with three clusters with different inter-cluster distances. All three methods show clear separation of the distant to the close clusters, with some separation between the two close clusters (Fig. 6). This is unexpected, as for t-SNE and UMAP, it is in contrast to the expectations of the authors, and literature [15, 19–21, 35]. Shepard diagrams and Pearson correlation analysis further show high to very high correlation between ambient and embedding distance for all methods (Fig. A14). This further strengthens the point that, contrary to common assumption, all methods are capable of creating embeddings preserving relative distance in addition to topography preservation, even for constructed edge cases meant to be hard to project. We believe this might be a result of the high dimensionality and non-uniform distribution around cluster centers of the fingerprints when compared with the synthetic datasets. Further analysis of the emergence of this phenomenon is warranted, but beyond the scope of this paper.

At closer inspection we can see a gap in projection distance between similar compounds with high Tanimoto similarity and dissimilar compounds with low overall similarity. This is especially pronounced when comparing UMAP and ESOM Shepard diagrams. One explanation for this can be how UMAP draws apart dissimilar clusters in embedding space, as seen in Fig. 6b. ESOM, however, can cluster distant points next to each other, with the U-distance only increasing monotonically at a maximum rate of 1 per neuron, as the U-distance between two adjacent neurons cannot be larger.

Inspection of the k-NN retention at different values of *k* (Fig. A15) reveals a slight advantage to t-SNE and ESOM at small values of *k* that diminishes and lead to and advantage of t-SNE and UMAP over ESOM at larger values.

Without rigorous statistical validation, we interpret the results of both analyses only as in indicator of non-inferiority of any of the method within the tested environment. However, further analysis akin to the analysis performed by Orlov et al. [40] is out of the scope of this work, but would be warranted.

Embeddings generated at *perplexity*/*n_neighbors* of 30, with otherwise default hyperparameters.

### Applicability of the ESOM algorithm to large cheminformatic datasets

Using χ-SOM, we were able to successfully train an ESOM ~ 5.5bn weights and ~ 1.7m datapoints within the span of 21 days. To our knowledge, this is the first time an ESOM of this size was trained; previous references to map size point to normal SOM with similar dataset sizes, but many fewer neurons and much smaller input vectors [23, 51].

The overview of the entire ChEMBL33 ESOM (Fig. A14) shows that both fluoroquinolone and beta-lactam antibiotics occupy distinct regions of the map, with individual outliers, discussed below. From pairwise similarity comparisons in Fig. 7, four distinct groups with high Tanimoto similarity become apparent. The first group comprises compounds 1–19, the second group compounds 20–39, the third group compounds 40–49, and the fourth group compounds 50 and 51. Compound 52 appears to be solitary. These groups align well with the distinct subtypes of beta-lactam antibiotics (BLA) that can be established from the compound depictions in Fig. A15. Group 1 corresponds to the penam subtype of BLA; group 2 corresponds to the cephem subtype; group 3 corresponds to the penem and carbapenem subtypes. Group 4 and compound 52 are of the monobactam subtype. Compound 52’s solitary position can be explained by the small influence the single beta-lactam ring has on the overall structural similarity of a relatively large molecule.

**Fig. 7:**
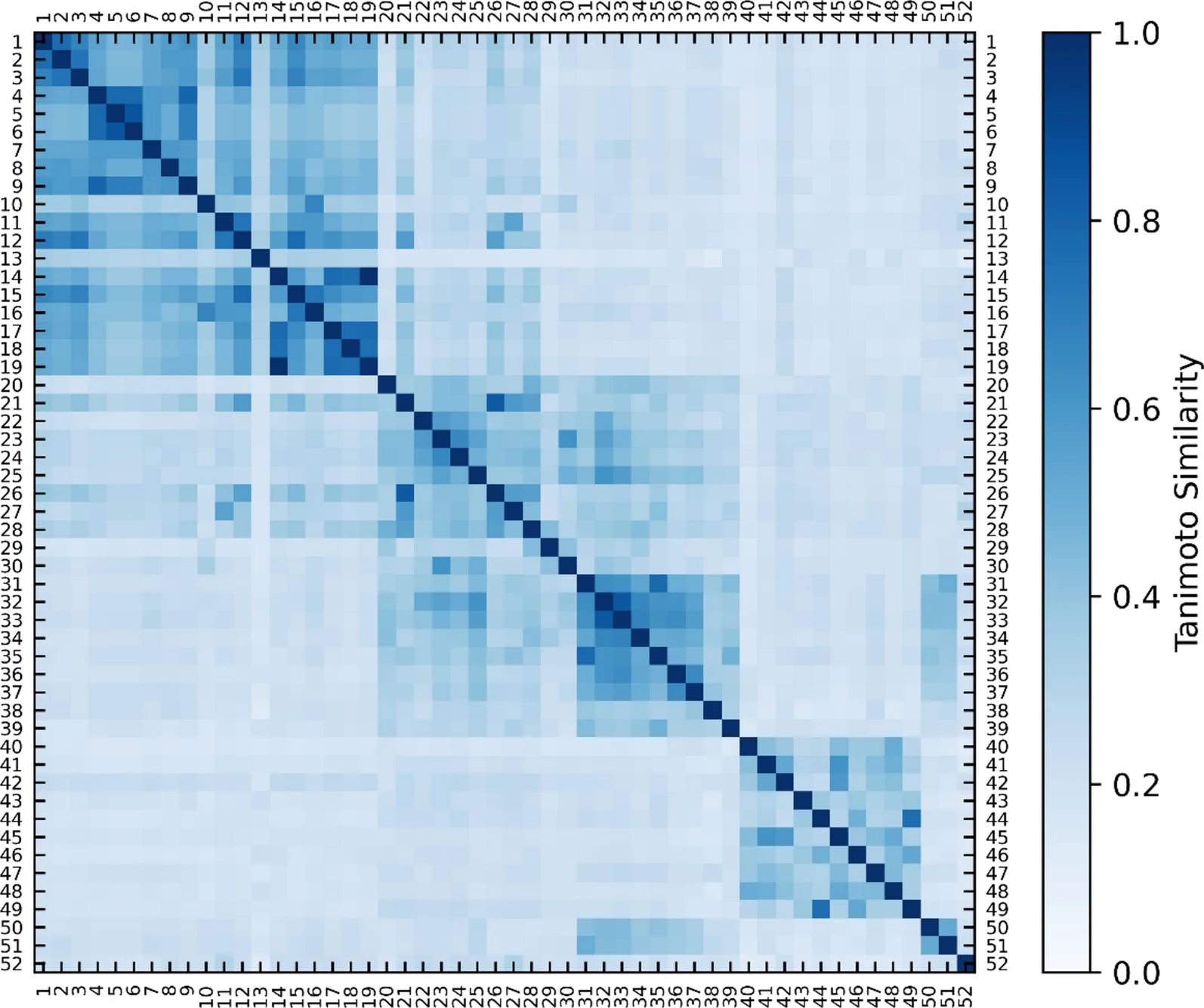
Pairwise similarity relationships of selected beta-lactam antibiotics, with the interaction patterns showing sub-types, including penams (1–19), cephems (20–39), penems (40–49), and monobactams (50–51)

In contrast, compounds 50 and 51 also share an aminothiazole and a further substituent at the ketoxime, after the amide bond. In the ESOM depiction of these similarity relationships in Fig. 8, direct correspondences are evident. Here, compounds 50 and 51 are mapped closely together.

**Fig. 8:**
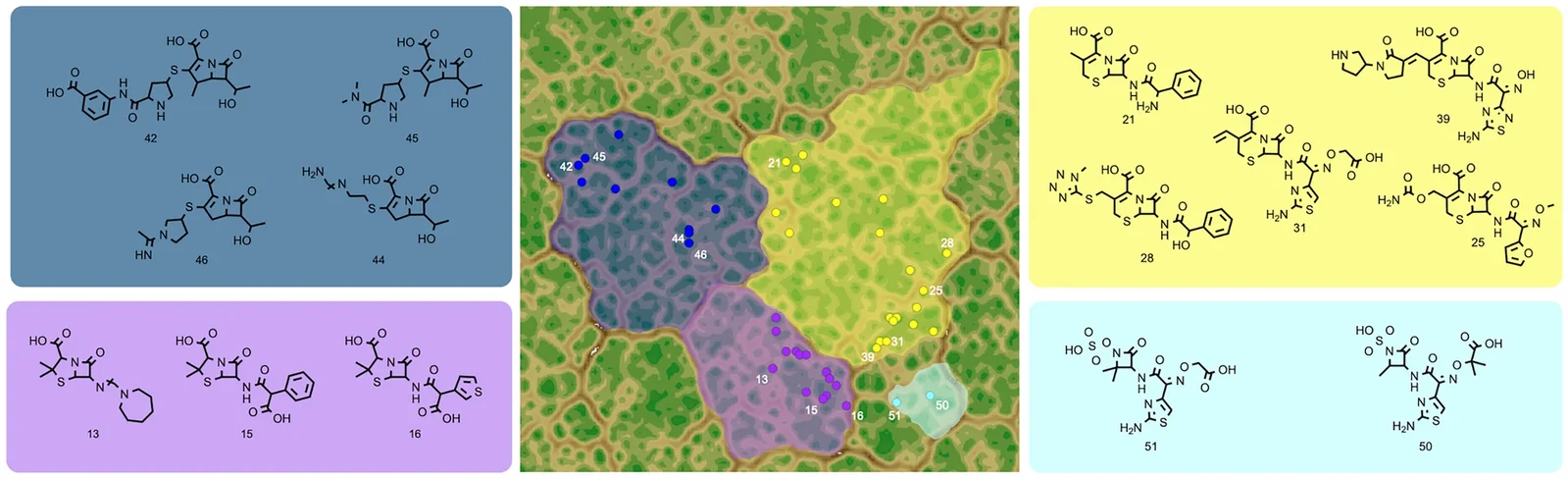
Image detail of the whole ChEMBL33 ESOM projection (Fig. A14) showing the space occupied by the beta-lactam antibiotics (BLA). Distinct subspaces of the BLA sub-types are indicated by different colors, with some exemplary compounds shown and their positions in the projection indicated by compound ID

Further investigation of the projection of all ChEMBL33 compounds reveals a larger area of monobactam compounds, colored in light blue. In a total view of the whole ChEMBL33 map, the outlier status of compound 52 becomes visible, with the compound being mapped to a completely different region; however, on further investigation, this is likely a poor match too, as indicated by the relatively high quantization error of 15.3 and very dissimilar projected ChEMBL33 compounds. An explanation might be that compounds similar to compound 52 are absent from this version of ChEMBL33. Given the low Tanimoto similarity, we conclude that this is not a projection error but rather a consequence of the chosen representation.

Investigation of the BMUs for compounds 1–19 shows that they are mapped closely together. Further analysis of all compounds from the ChEMBL33 dataset projected to this area reveals a larger subspace of penam-type beta-lactam antibiotics (purple-colored, Fig. 8) that is well separated from other regions of the map, as indicated by ridges of large U-height surrounding it.

Closer inspection reveals even finer-grained subspaces within. Compounds 14, 15, 17, 18, and 19, which show high similarity and all contain a benzene ring at the same position, are mapped to two directly neighboring neurons. Compound 16, which shares only partial similarity due to the thiophene ring rather than the benzene ring, is mapped slightly farther to the southeast. It shares this BMU with compound 10, which is of the same structure, however with a methoxy group directly attached to the beta-lactam ring, likely changing the fingerprint of the whole structure significantly, as seen in the light interaction pattern in Fig. 7. This analysis also holds for further interaction patterns visible, including the patterns for compounds 4–6, 11–12, 7–9.

The similarity pattern for cephem compounds is less pronounced overall. This property is also reflected in the ESOM projection, as the corresponding BMUs are spaced farther apart on the map. Compounds 32–39 form a group that shows a stronger interaction pattern and all contain an aminothiazole ring at the same position. Compound 31, in which the aminothiazole ring is replaced by a thiadiazole ring, is projected in close proximity. They also show an interaction pattern with the monobactams 50 and 51, likely due to the shared substructure from the thiazole ring to the beta-lactam ring. They are also the cephem-group entities projected closest to the monobactams on the ESOM.

A little further removed, compound 25 can be found, with an overall similar structure but with the nitrogen-containing heterocycles replaced by a furan at the same position. Even though compounds 21, 26, and 27 show a high overall similarity to compound 28, compound 28 is projected to a distant neuron on the map. This is likely due to the influence of the large substituent at position 3. When investigating all compounds from the training set projected into this area, it becomes clear that compound 28 is indeed part of a set of compounds with similar substituents at position 3, forming a distinct region in ambient space.

Investigating all compounds in the ChEMBL33 projection that fall into the same area and following the ridge separating the embedding space, a distinct subspace for the cephem group becomes apparent (yellow background), well separated from other drugs by a large U-height and from other beta-lactams by ridges of slightly lower U-height.

Comparing the interaction patterns of the penem subtype 40–49 with the ESOM projection, again, a less dense but distinct subspace becomes visible (dark blue), contrasted against the space occupied by other drugs and other subtypes of beta-lactam antibiotics, as indicated by ridges of different U-height. Interaction patterns, such as those among compounds 44, 46, and 49, are reflected in smaller distances on the ESOM projection between the more similar compounds. We also observe differing substitution patterns that lead to subclusters while preserving overall proximity on the map between compounds such as 42 and 45. Here, the large substituent in compound 42 leads to a pronounced ridge between it and compound 45.

Similar observations apply to the gyrase-inhibiting type of antibiotics (Fig. A16/A17/A18). For example, Fig. A17 shows a high degree of similarity among compounds 1, 3, and 10. This can be attributed to the methylenedioxy substructure, which is shared only by these three compounds and is reflected in their proximity on the ESOM map in Fig. A18. The outlier in the ChEMBL33 map overview is compound 6, which shows low overall similarity to the other compounds. Analysis of the ChEMBL33 compounds projected to the same area shows that they are indeed very similar to compound 6.

Shepard diagram and spearman correlation analysis reveal nearly no correlation between ambient and embedding distance (see Fig. A21). Most datapoint pairs show low values of Tanimoto similarity in combination with heavily varying normalized u-distance. Additionally, a tail of very small u-distance ranging from low values of Tanimoto similarity up to 1 is visible. However, we believe this distinct “L”-shaped distribution is an artifact of the distance measures used in combination with the map size and not an indicator of poor method performance. Tanimoto similarity strongly depends on the number of same on-bits set. With few to no on-bits in common, Tanimoto similarity only varies at the very low end of the scale, aligning well with the small average Tanimoto similarity across all compound pair in Fig. A22. At the same time, the number of non-matching bits between fingerprints, can still convey varying dissimilarity not captured by the measure. This can lead to distinct compounds with completely different scaffolds and features only exhibiting small variations in Tanimoto similarity. However, they will be embedded in vastly different areas of the map, that especially at this size of ESOM. This will lead to greatly varying u-distance for most compounds, while normalization will squash u-distances between close pairs to nearly zero. In combination with our sampling strategy this leads to the distribution visible with the large cloud of small Tanimoto similarity but greatly varying normalized u-distance and the thin tail of very small u-distance for most larger values of Tanimoto similarity. Similarity measures other than Tanimoto similarity might remedy this to some extent, however they too suffer a loss of expressiveness with large distances between high dimensional objects, often termed “curse of dimensionality” [52].

Inspection of the k-NN retention analysis (Fig. A23) shows neighbor retention significantly better than chance, with a slight drop at high values of *k*. Retention is also seemingly poorer than on the previous, constructed, datasets. This reduced performance in comparison however is to be expected with an increase in the number of compounds, as the complexity of the topology increases, fundamentally making the creation of a continuous embedding harder.

## Conclusion

Our results indicate that ESOMs, as implemented in χ‑SOM, are a viable and currently underused alternative to dimensionality-reduction methods such as t-SNE and UMAP in the visual exploration of chemical space. On synthetic benchmarks probing density and inter‑cluster distance preservation, ESOM retained information that was only partially or not at all reflected in t‑SNE and UMAP embeddings across the range of hyperparameters we examined. In particular, ESOM captured differences in cluster densities and inter-cluster distances in a manner that remained stable as the dataset size increased. In contrast, t-SNE and UMAP homogenized cluster spacing and failed to reflect these properties reliably. The combination of a high-resolution neuron grid with the U-Matrix allowed clusters and separating ridges to emerge in a manner that provides a notion of both direction and relative distance in the underlying ambient space.

When analyzing the same set of problems represented as cheminformatic fingerprints, this advantage diminishes. All three methods show strong performance on both the density and inter-cluster distance benchmark sets. This is in contrast with literature on the topic, where it is often assumed that both t-SNE and UMAP fail to preserve these features. However, these claims are often only supported by synthetic datasets, similar to the ones used herein. We note that this comparison is not a systematic hyperparameter optimization for any of the three methods, and that ESOM is itself sensitive to hyperparameters we held fixed here, in particular the neuron count, the boundary condition, and the map aspect ratio. The stability we report should therefore be read as stability across the hyperparameters we varied, and not as a general claim. These observations warrant future research, which is beyond the scope of this paper.

Two features remain that stand out as advantages of ESOM. First, ESOM tends to be more stable to hyperparameter variation, where t-SNE and UMAP are sensitive to *perplexity*/*n_neighbors* settings in relation to the number of datapoints and the hidden structure within the dataset with regard to cluster size. While the number of datapoints can easily be established a priori, the hidden cluster size cannot. In ESOM, the number of neurons hyperparameter also needs to scale with the number of datapoints, however the heuristics proposed by Ultsch et al. always seem to generate reasonable results under those assumptions. Furthermore, ESOM does not show crowding of nearby datapoints under this heuristic. For all datapoints both the local and global similarity relationships can be established using a single projection.

From a practical perspective, χ-SOM addresses scalability limitations that have hindered the wider use of SOM-based navigation in chemical space. Benchmarking against a diverse set of existing SOM libraries showed that our GPU-accelerated online implementation achieves competitive or superior runtimes for dense cheminformatic fingerprints while avoiding the memory bottlenecks found in other implementations. While sparse implementations, such as sparse-som, can outperform χ-SOM in runtime for sparse fingerprints, this comes at the cost of limited features (e.g., the set of distance metrics) and reduced performance on dense representations. χ-SOM instead provides a solution that supports multiple distance measures, delivers high performance on a wide range of compute hardware, and offers tools for visual investigation of ultra-large libraries of chemical fingerprint representations.

We conclude with an investigation into both our implementations and the ESOM architecture’s applicability to large scale cheminformatic datasets. At 5.1 million neurons, we created what is presumably the largest ESOM to date using our χ-SOM implementation. We used projections of both beta-lactam and gyrase-inhibiting antibiotics to investigate their projection properties, with a specific focus on how well they align with a medicinal chemist’s view of chemical similarity.

Beyond the Shepard diagram with correlation analysis, showing mixed results, and k-NN retention analysis in Fig. A15 and A15 the comparison presented here rests on visual inspection of the embeddings. We believe that only a rigorous, statistical investigation, including the u-distance, would result in a meaningful measurement of the ESOM embedding capability. Currently, however, even at the lower end of acceptable sampling of datapoints, using this analysis is computationally infeasible. Significant engineering work is necessary to enable such analysis. Given the positive results of the k-NN retention analysis, in combination with the results of the medicinal chemist’s assessment, ESOM and in particular our χ-SOM implementation, can be seen as a valuable tool for chemical space analysis.

We see potential to further expand this framework by rigorously evaluating ESOM for cheminformatic use cases and by enhancing the capabilities of the χ-SOM library itself.

In addition to applying our χ-SOM implementation to ongoing and future drug discovery projects, we also see potential to evaluate the capabilities of ESOM in domains outside of chemical space analysis including visualization of protein–ligand interactions or molecular dynamics.

## Supplementary Information

Supplementary Material 1

Supplementary Material 2

## Data Availability

Project name: χ-SOM. Project home page: https://github.com/kochgroup/ChI-SOM. Operating system(s): Linux. Programming language: Python. Other requirements: Python 3.13, CUDA-enabled GPU with compute capability 5.0 or higher for GPU compute. License: GNU LGPL. Any restrictions to use by non-academics: None. Data and Materials, as well as the versions of χ-SOM used during the investigation, are available at https://doi.org/10.17879/5110d-qdn06.

## Abbreviations

SOM: Self-Organizing Map
ESOM: Emergent Self-Organizing Map
t-SNE: T-distributed Stochastic Neighbor Embedding
PCA: Principal Component Analysis
UMAP: Uniform Manifold Approximation and Projection
k-NN: K-Nearest-Neighbor
GUI: Graphical User Interface
BMU: Best-Matching Unit
BLA: Beta-Lactam Antibiotic
DR: Dimensionality Reduction
