# Supplementary material for: χ-SOM: emergent self-organizing maps for large chemical space visualization
Source: J Cheminform. 2026 Oct 18;18:150. doi: 10.1186/s13321-026-01317-z (PMC13721295; doi:10.1186/s13321-026-01317-z)
Supplement: Supplementary file 1 [file 13321_2026_1317_MOESM1_ESM.pdf]

Supporting Information A

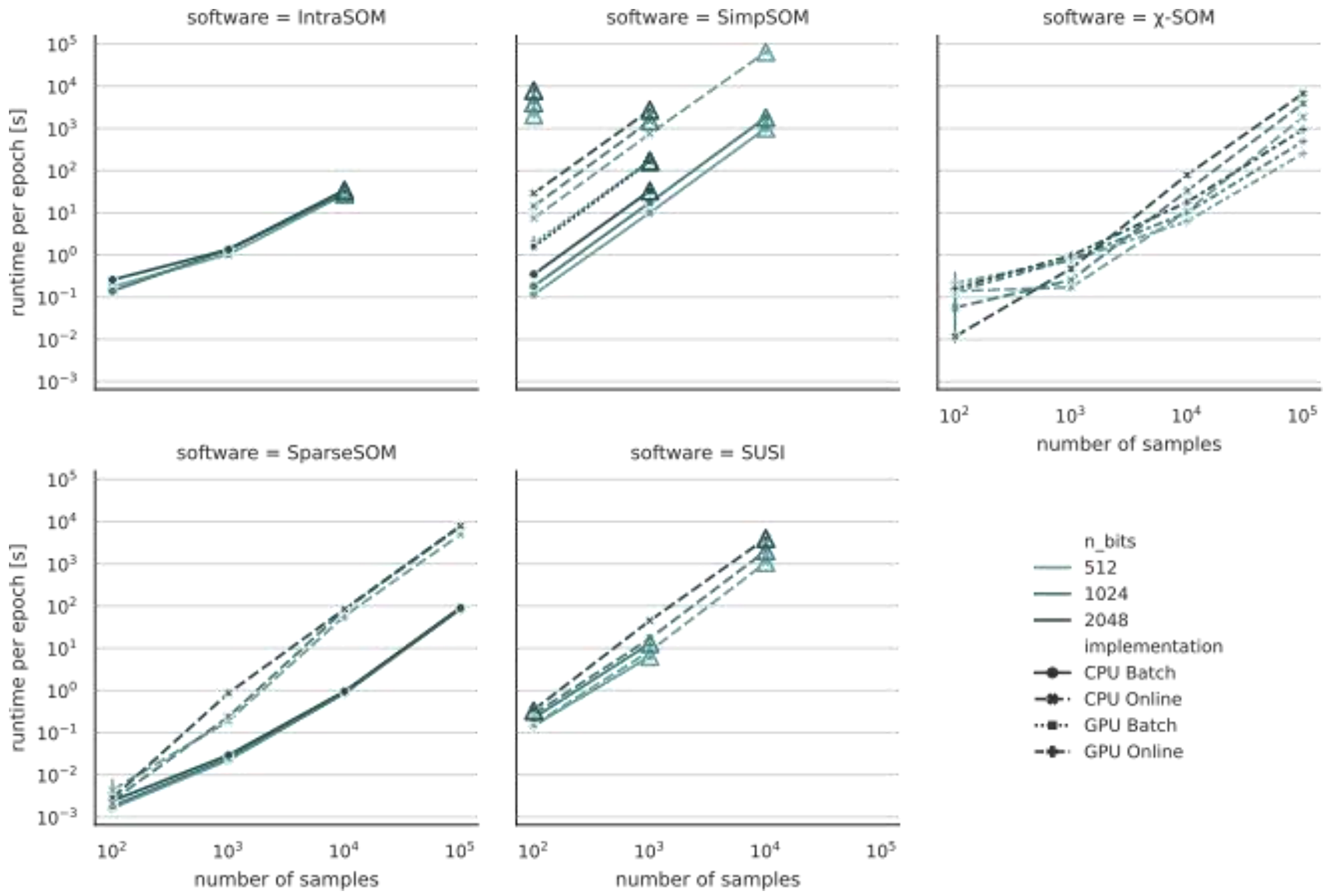

Figure A1: Runtime per epoch, in seconds, of different implementations of the SOM algorithm. Each value is the mean over 15 (three sampled datasets × five epochs) at identical parameter settings. Triangles mark the last measurable configuration; the next larger sample count exceeded the runtime or memory limit. Absolute error bars are shown, although the spread is often too small to be visible

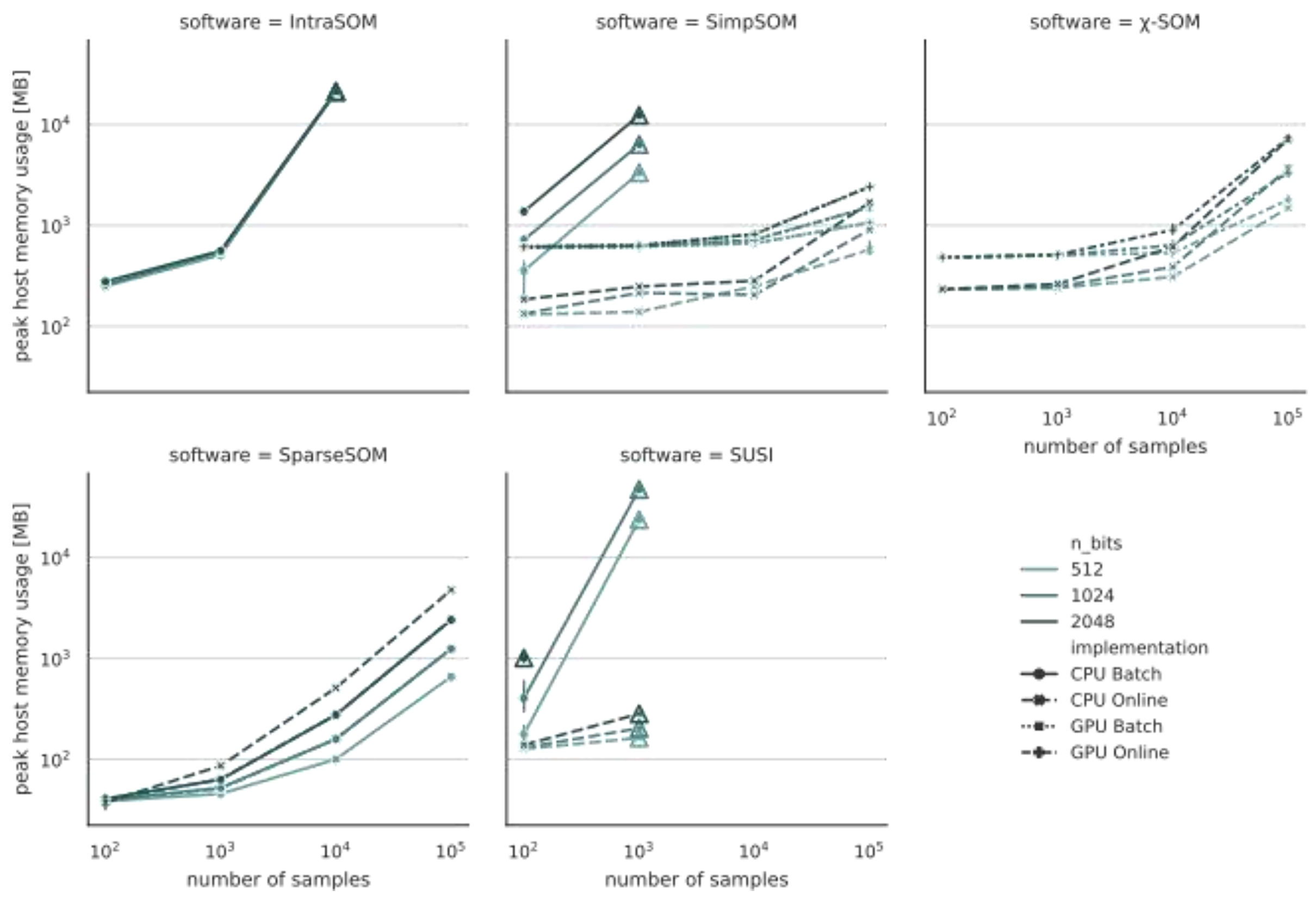

Figure A2: Host memory usage, in megabyte, of different implementations of the SOM algorithm. Each value is the mean over 15 (three sampled datasets × five epochs) at identical parameter settings. Triangles mark the last measurable configuration; the next larger sample count exceeded the runtime or memory limit. Absolute error bars are shown, although the spread is often too small to be visible

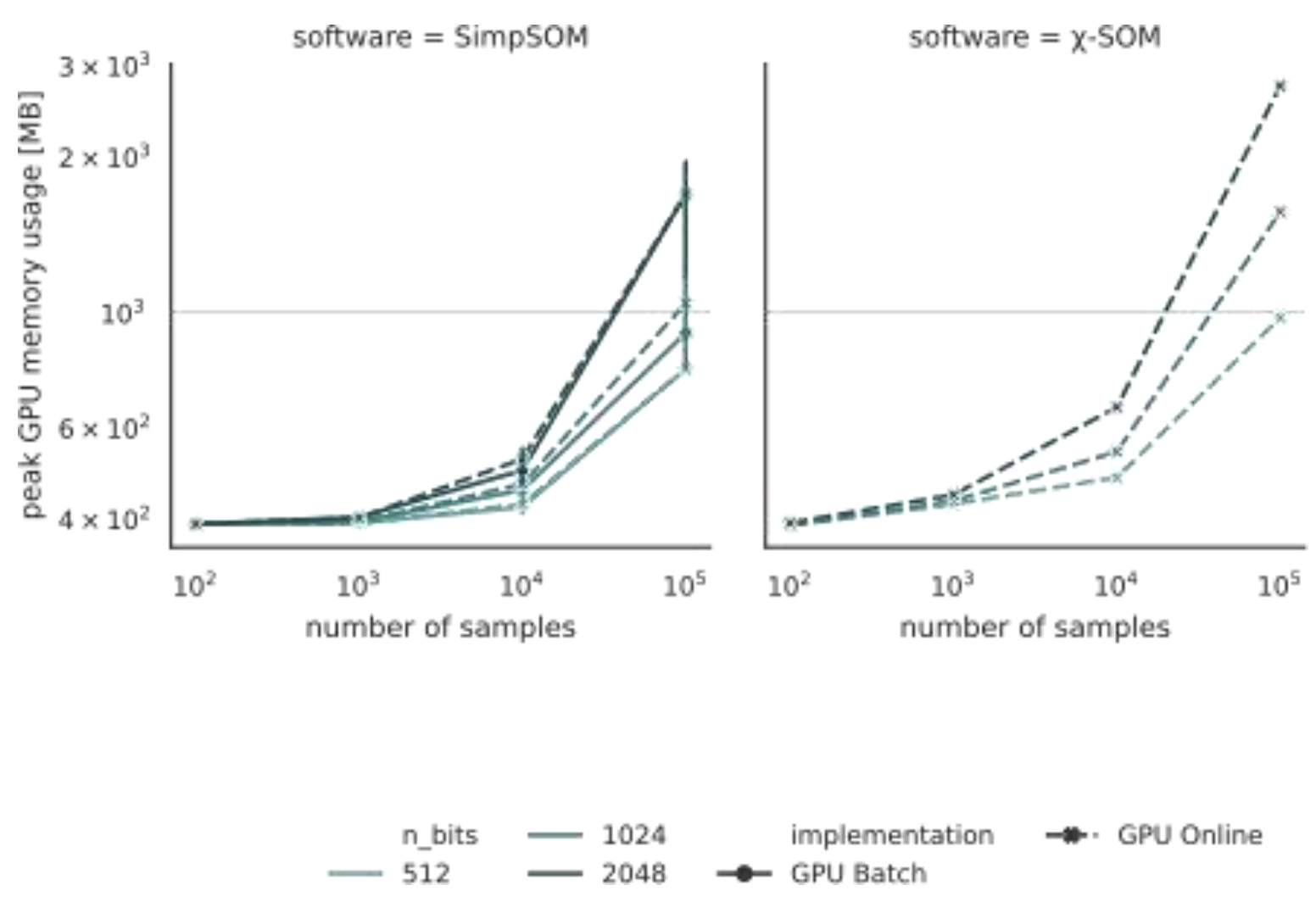

Figure A3: GPU memory usage, in megabyte, of different implementations of the SOM algorithm. Each value is the mean over 15 (three sampled datasets × five epochs) at identical parameter settings. Triangles mark the last measurable configuration; the next larger sample count exceeded the runtime or memory limit. Absolute error bars are shown, although the spread is often too small to be visible

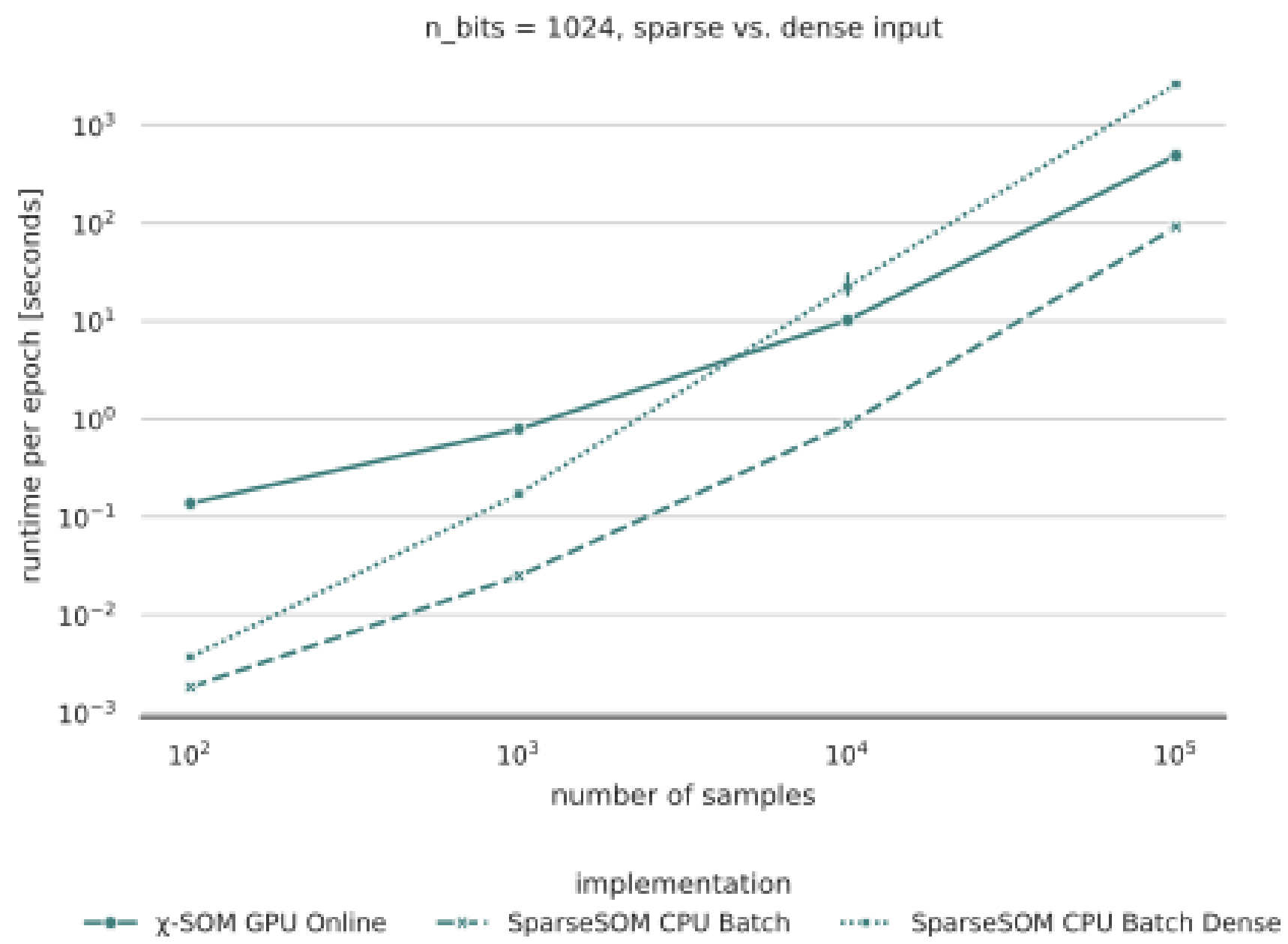

Figure A4: Runtime per epoch comparison of χ-SOM against the sparse-som CPU batch implementation on sparse and dense datasets.

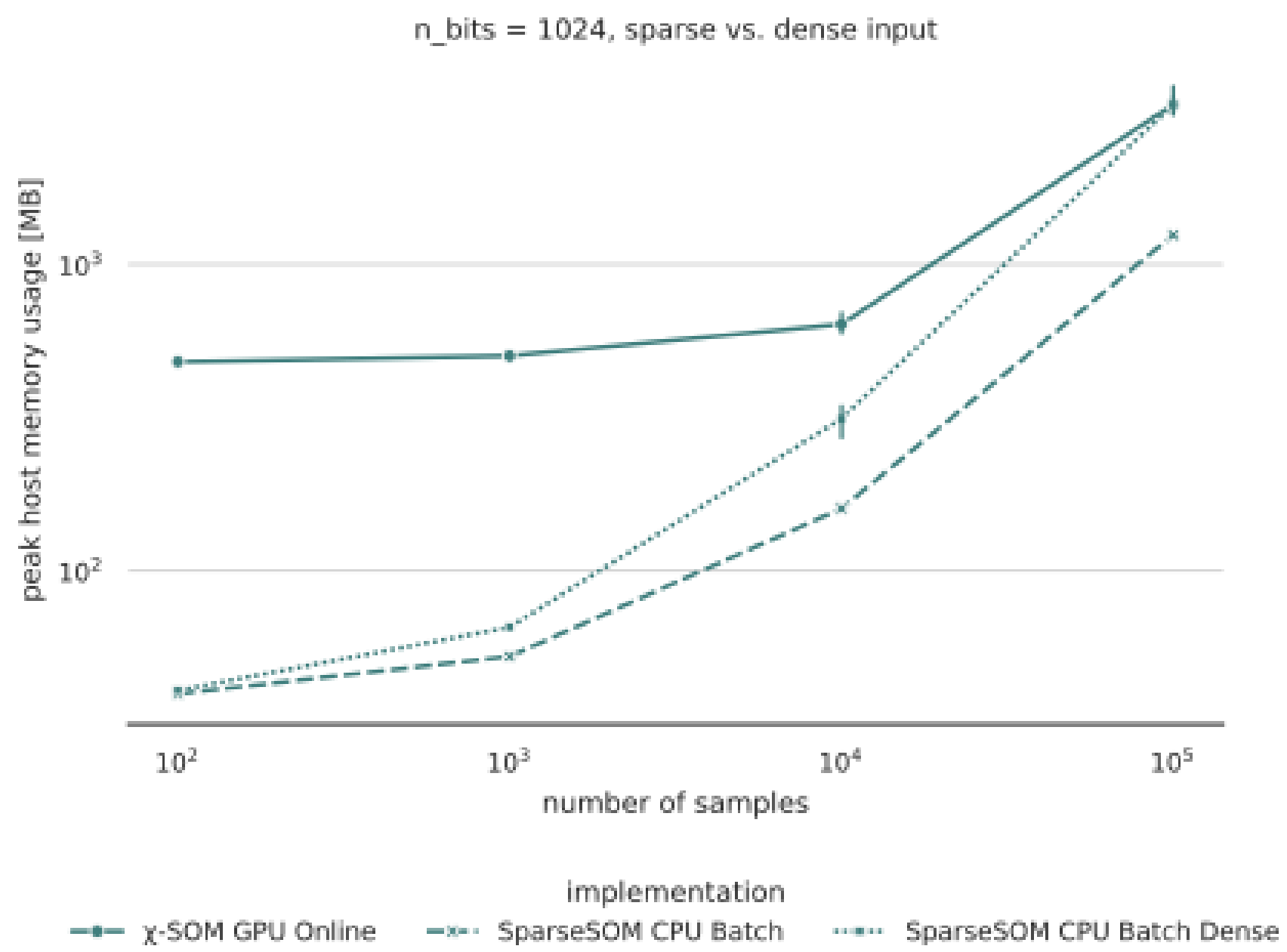

:

Figure A5: Host memory usage per epoch comparison of χ-SOM against the sparse-som CPU batch implementation on sparse and dense datasets.

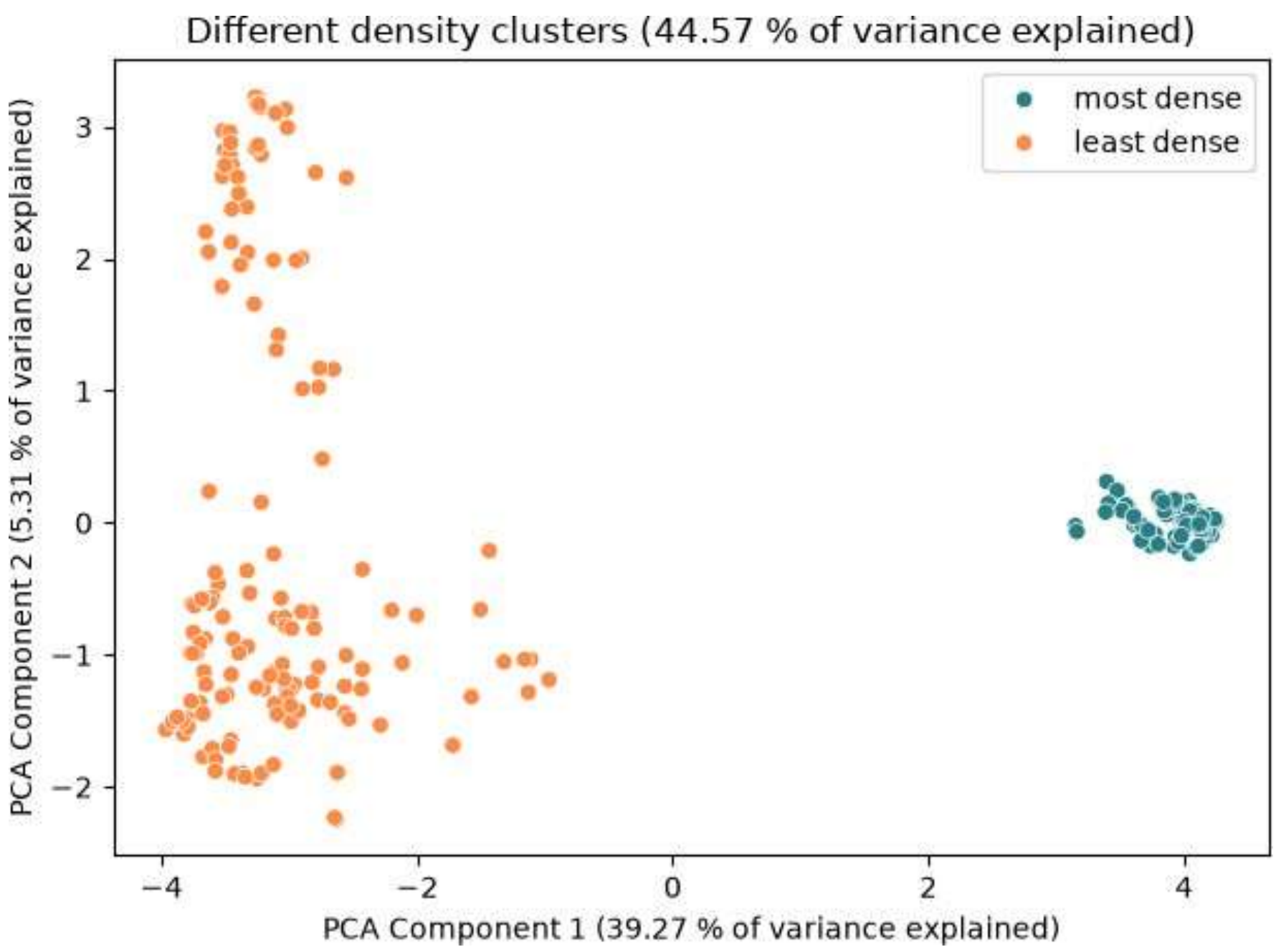

Figure A6: Plot of the first two principal components of the density cluster datasets constructed from ChEMBL33 using BitBIRCH-Lean.

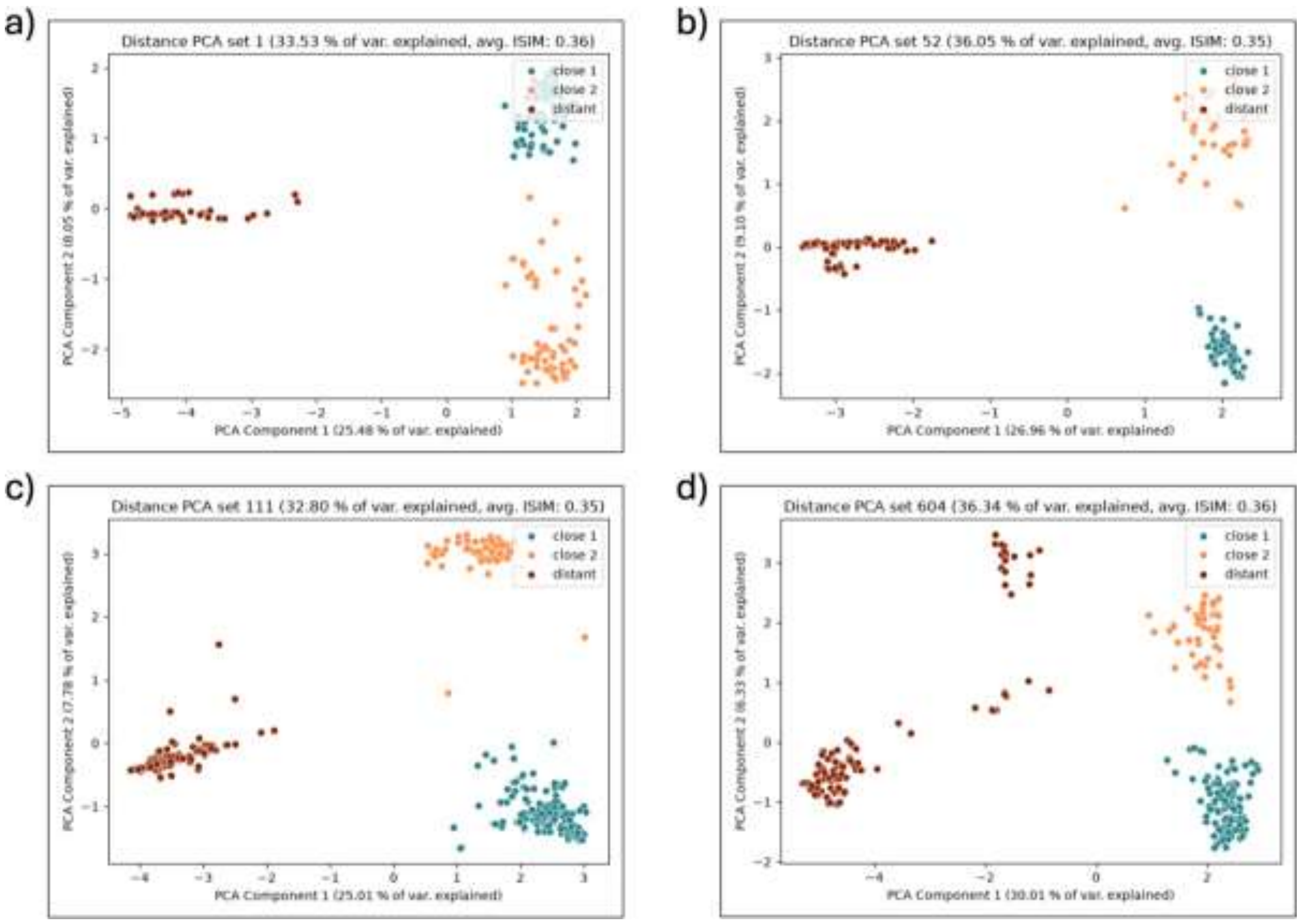

Figure A7: Plots of the first two principal components of the triangular distance relationship cluster datasets constructed from ChEMBL33 using BitBIRCH-Lean. Set 604 (reffered to as d604) is used in all distance-based assessments on fingerprints. All additional plots are available in Supporting Information B

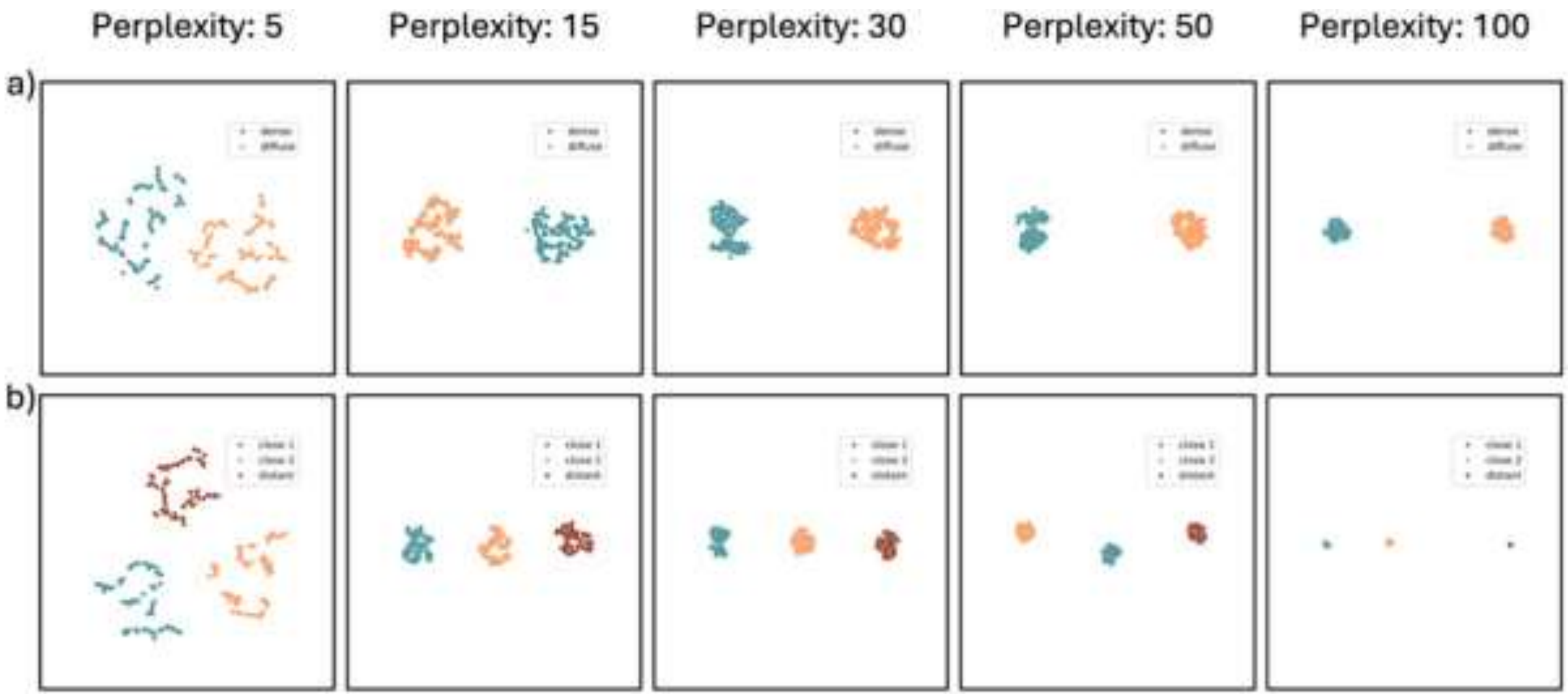

Figure A8: Plots of t-SNE embeddings of the synthetic datasets at different values of *perplexity*. Other parameters set to default settings. All additional plots are available in Supporting Information B.

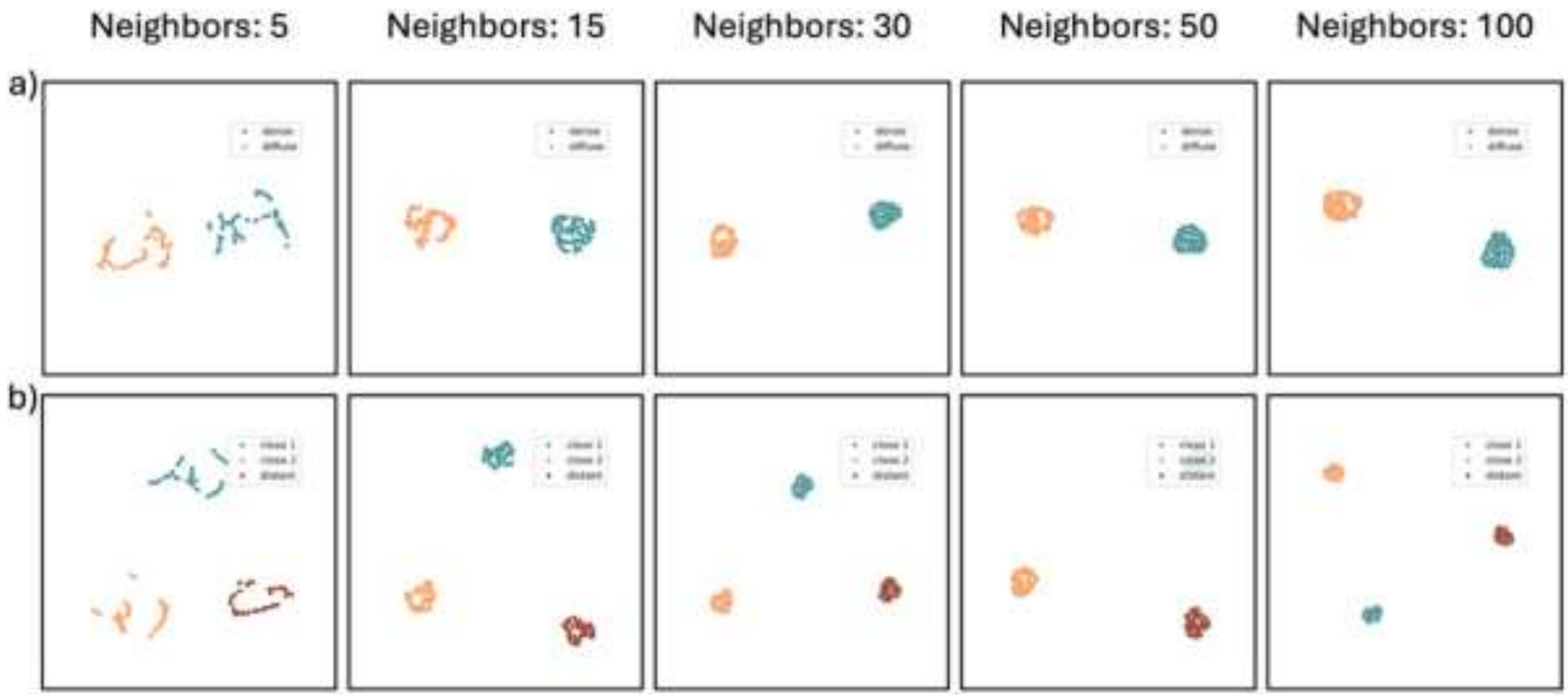

Figure A9: Plots of UMAP embeddings of the synthetic datasets at different values of *n_neighbors*. Other parameters set to default settings. All additional plots are available in Supporting Information B.

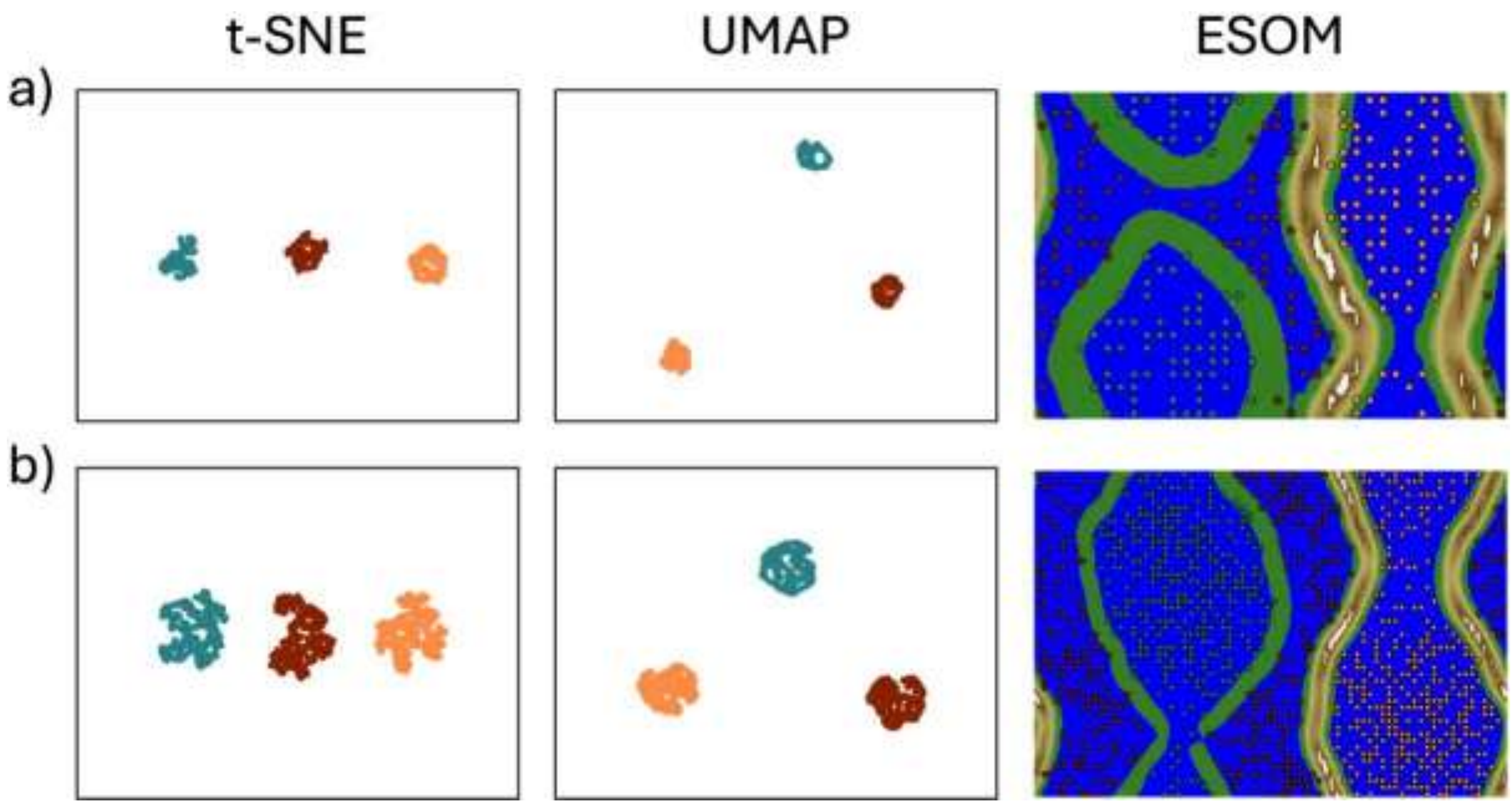

Figure A10: Plots for t-SNE, UMAP and ESOM projecting clusters with different inter-cluster distances at a) 100 data points per cluster and b) 200 data points per cluster.

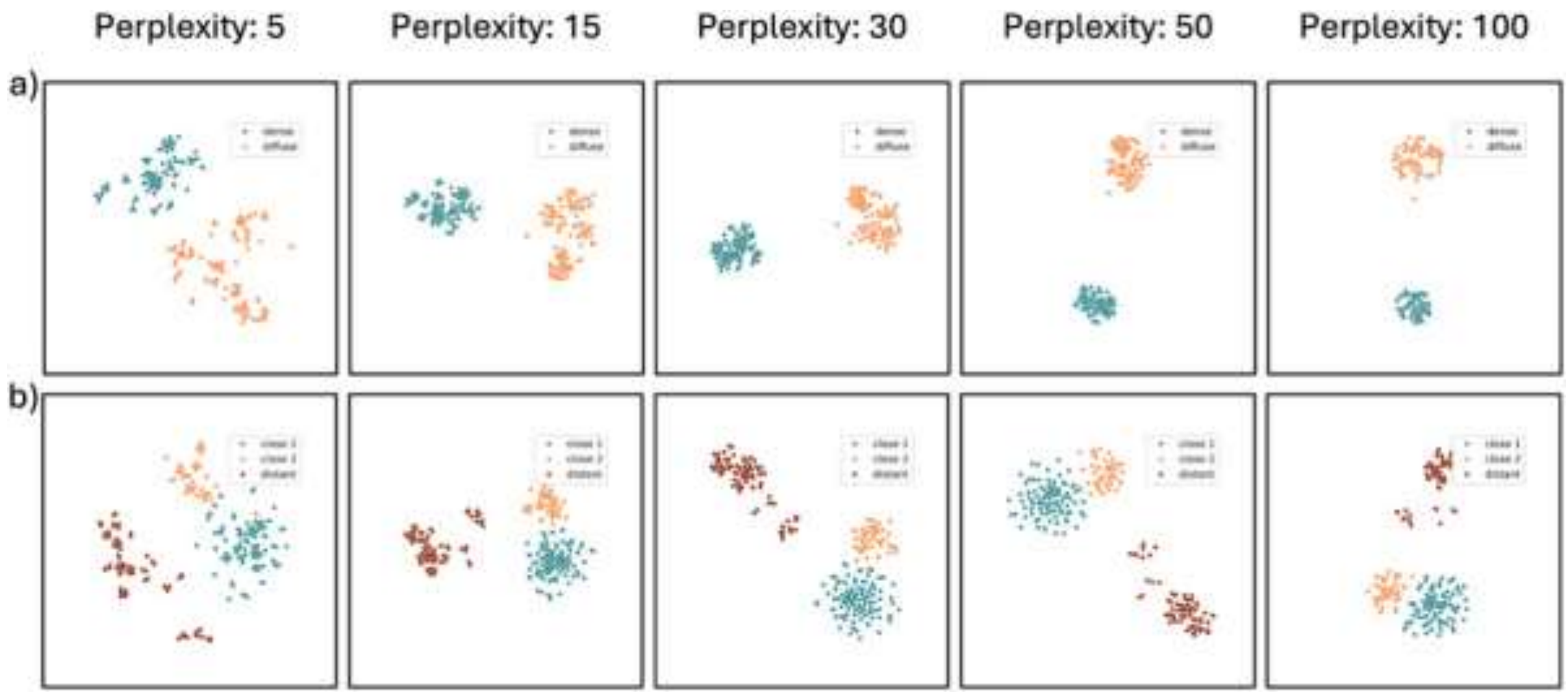

Figure A11: Plots of t-SNE embeddings of the a) density fingerprint datasets and b) inter-cluster distance dataset d604 at different values of *perplexity*. Other parameters set to default settings. All additional plots are available in Supporting Information B.

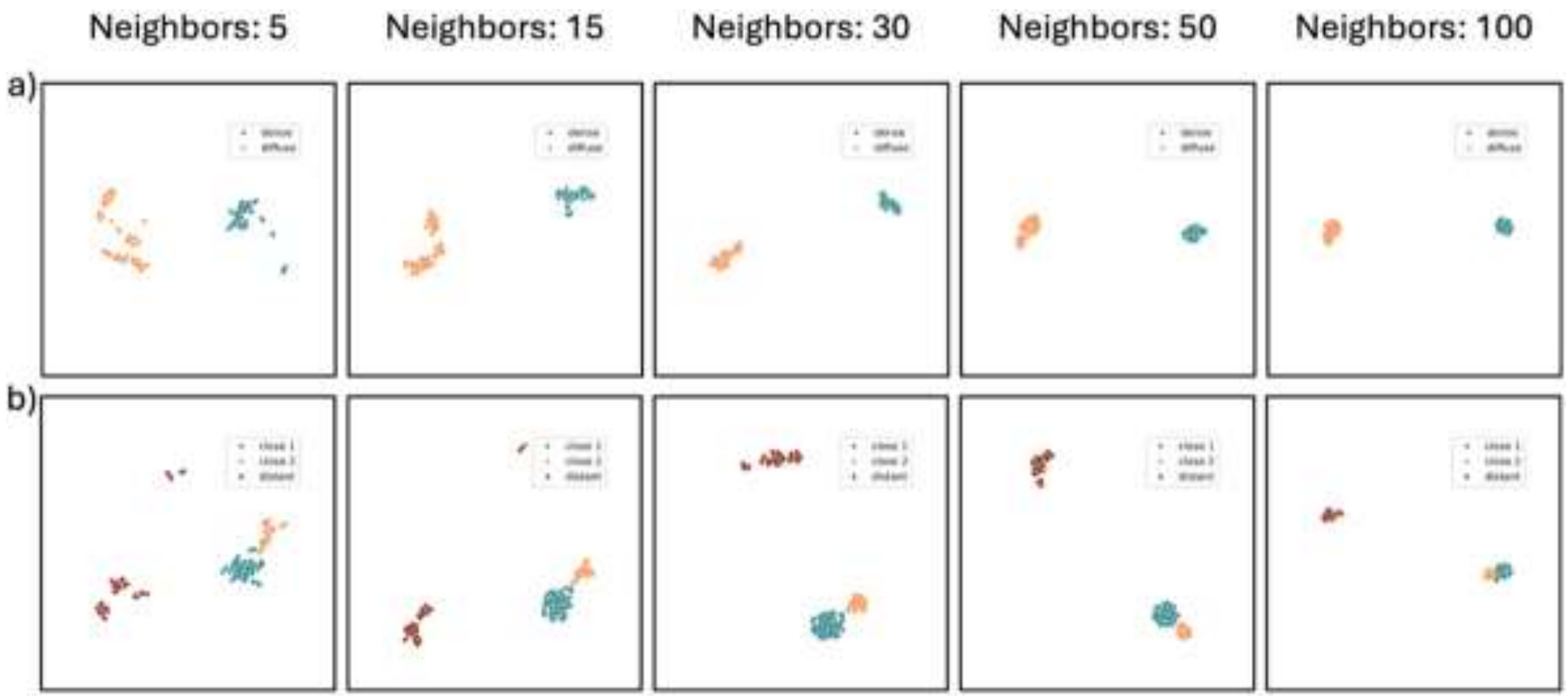

Figure A12: Plots of UMAP embeddings of the a) density fingerprint datasets and b) inter-cluster distance dataset d604 at different values of *n_neighbors*. Other parameters set to default settings. All additional plots are available in Supporting Information B.

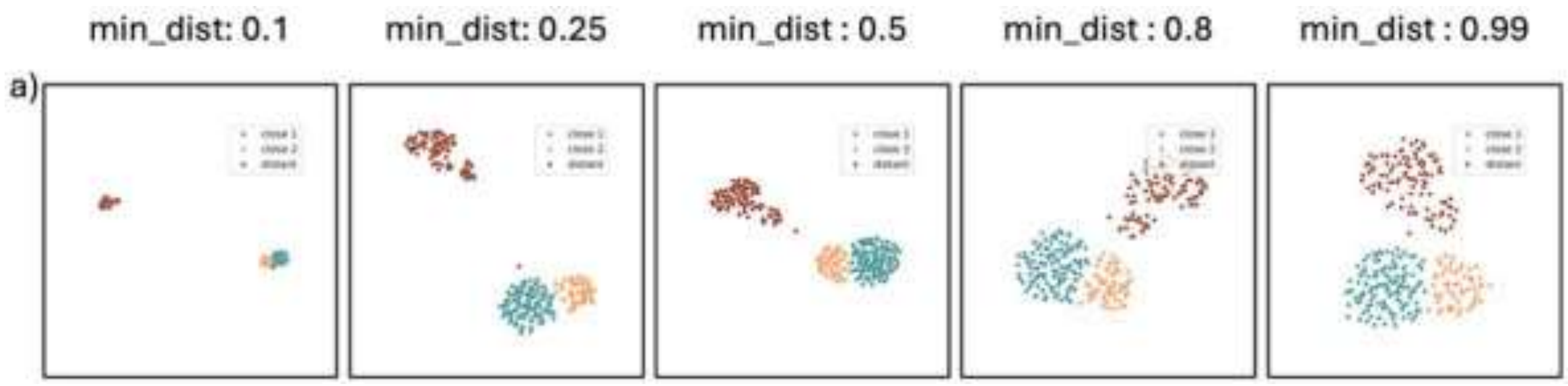

Figure A13: Plots of UMAP embeddings of the fingerprint datasets d604 at different values of *min_dist*. Other parameters settings like main Figure 6b. All additional plots are available in Supporting Information B.

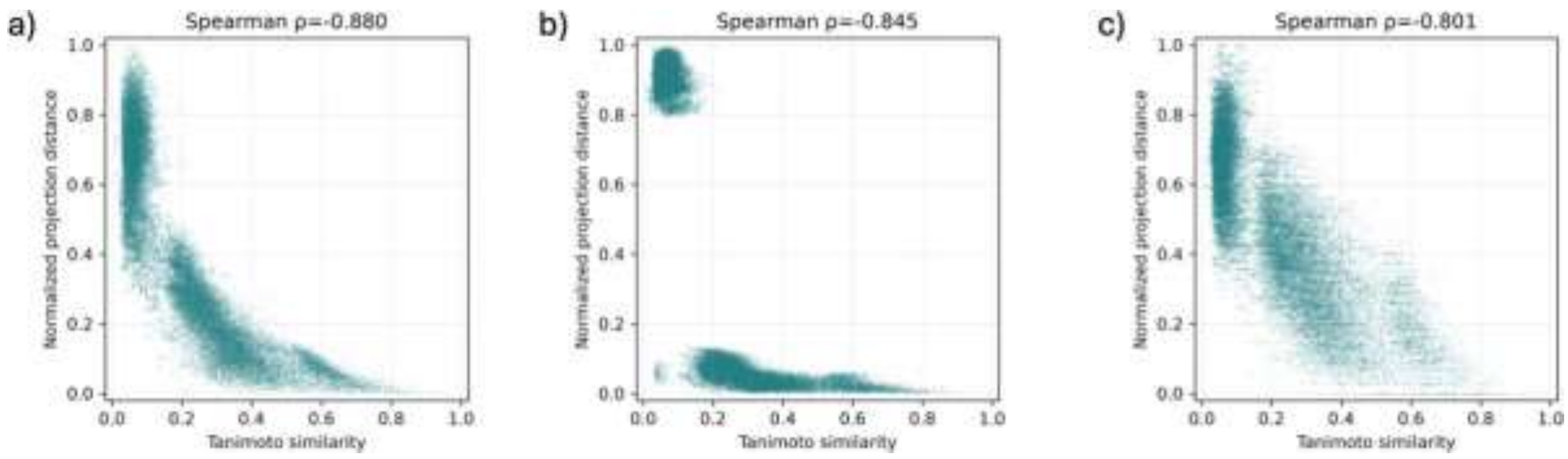

Figure A14: Shepard diagrams with Pearson correlation of the a) t-SNE, b) UMAP and c) ESOM embeddings shown in Fig. 6b. Projection distance was normalized using min-max-scaling.

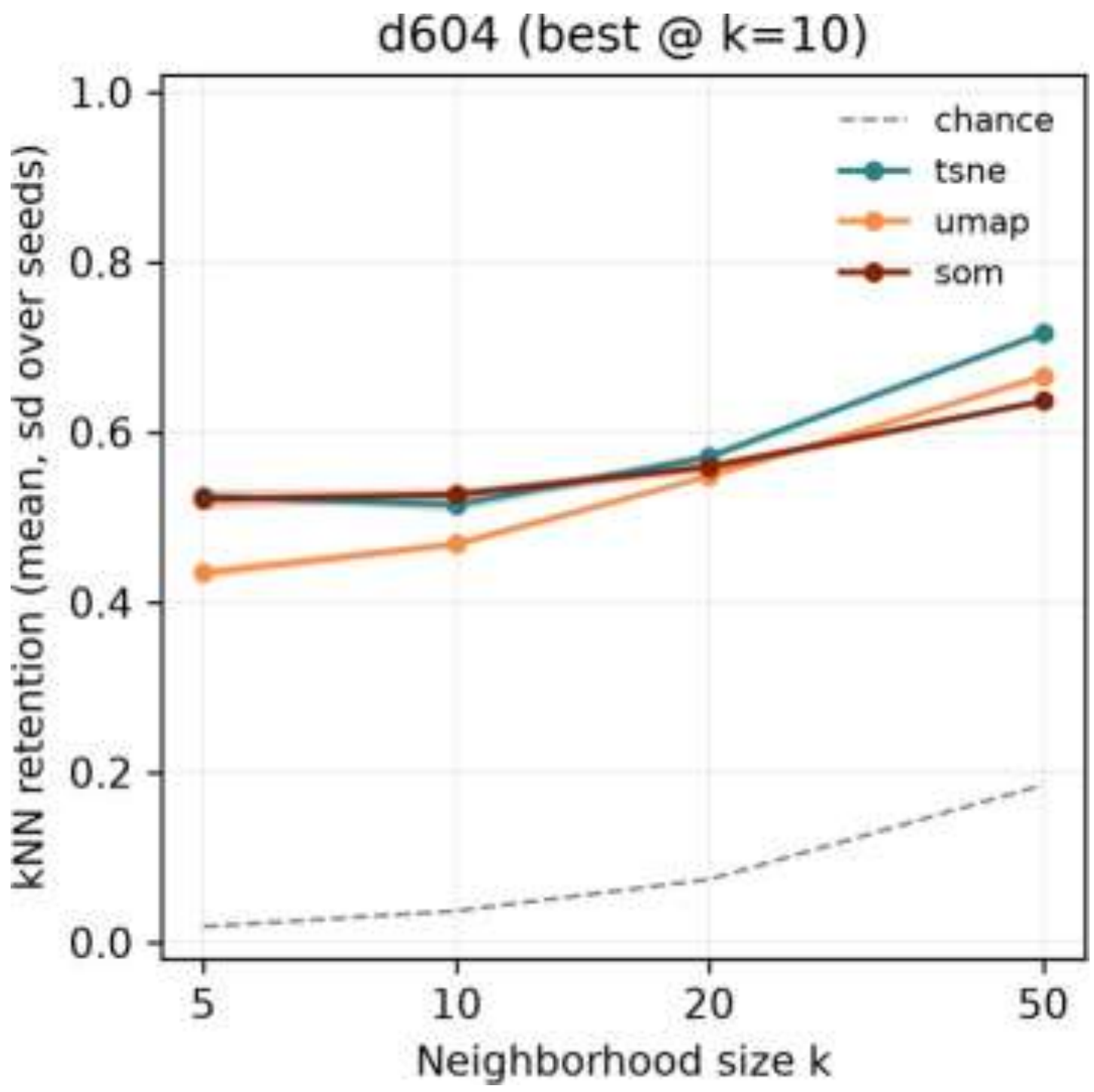

Figure A15: k-Nearest-Neighbor retention for all three methods at different values of k. Only the best performing hyperparameter combination on dataset d601 is shown. Dashed line shows the expected value if embeddings are place randomly.

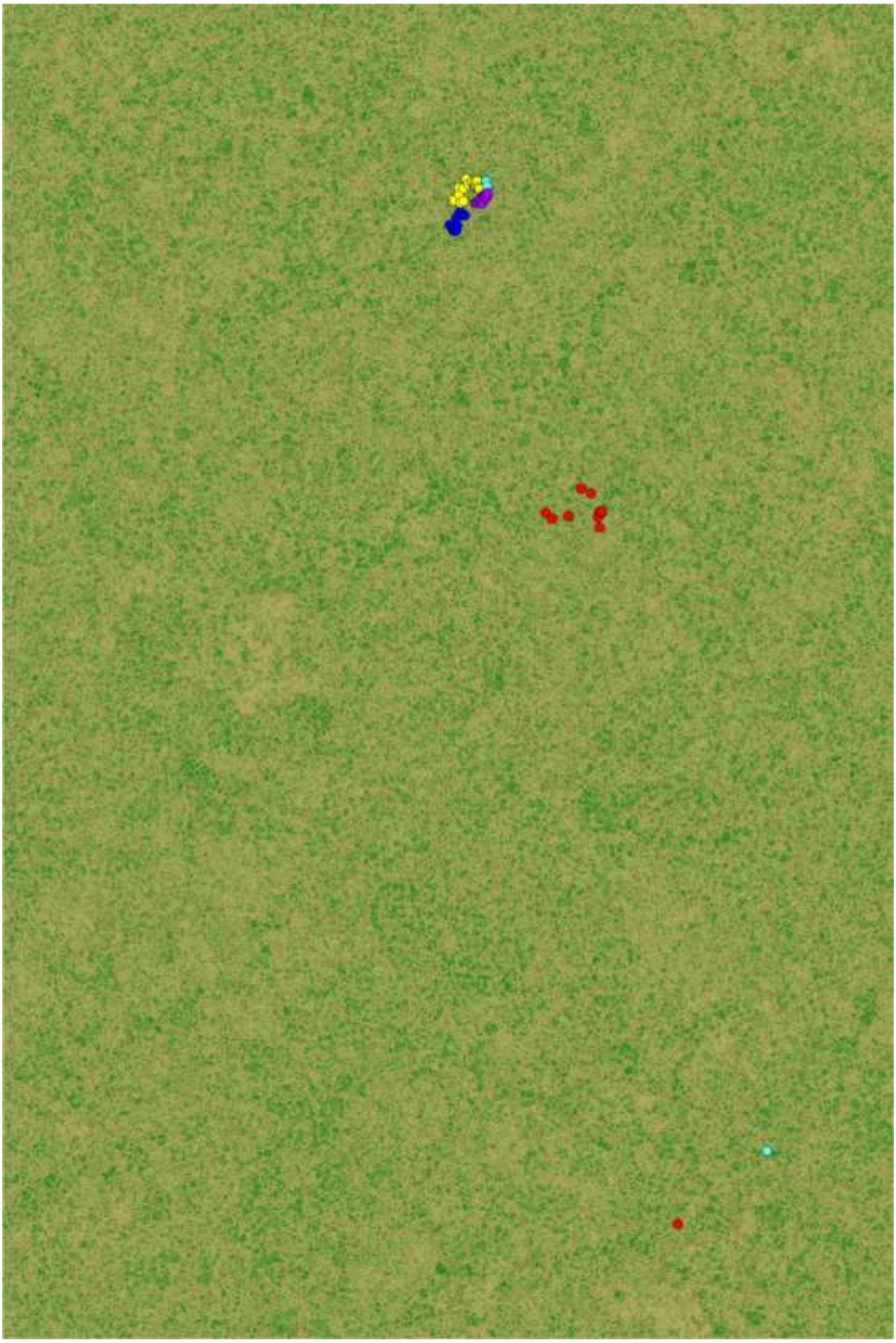

Figure A16: ESOM map of the complete ChEMBL33 dataset. Best-matching units (BMU) of the gyrase-inhibiting compounds are colored in red. BMUs of beta-lactam antibiotics are colored blue.

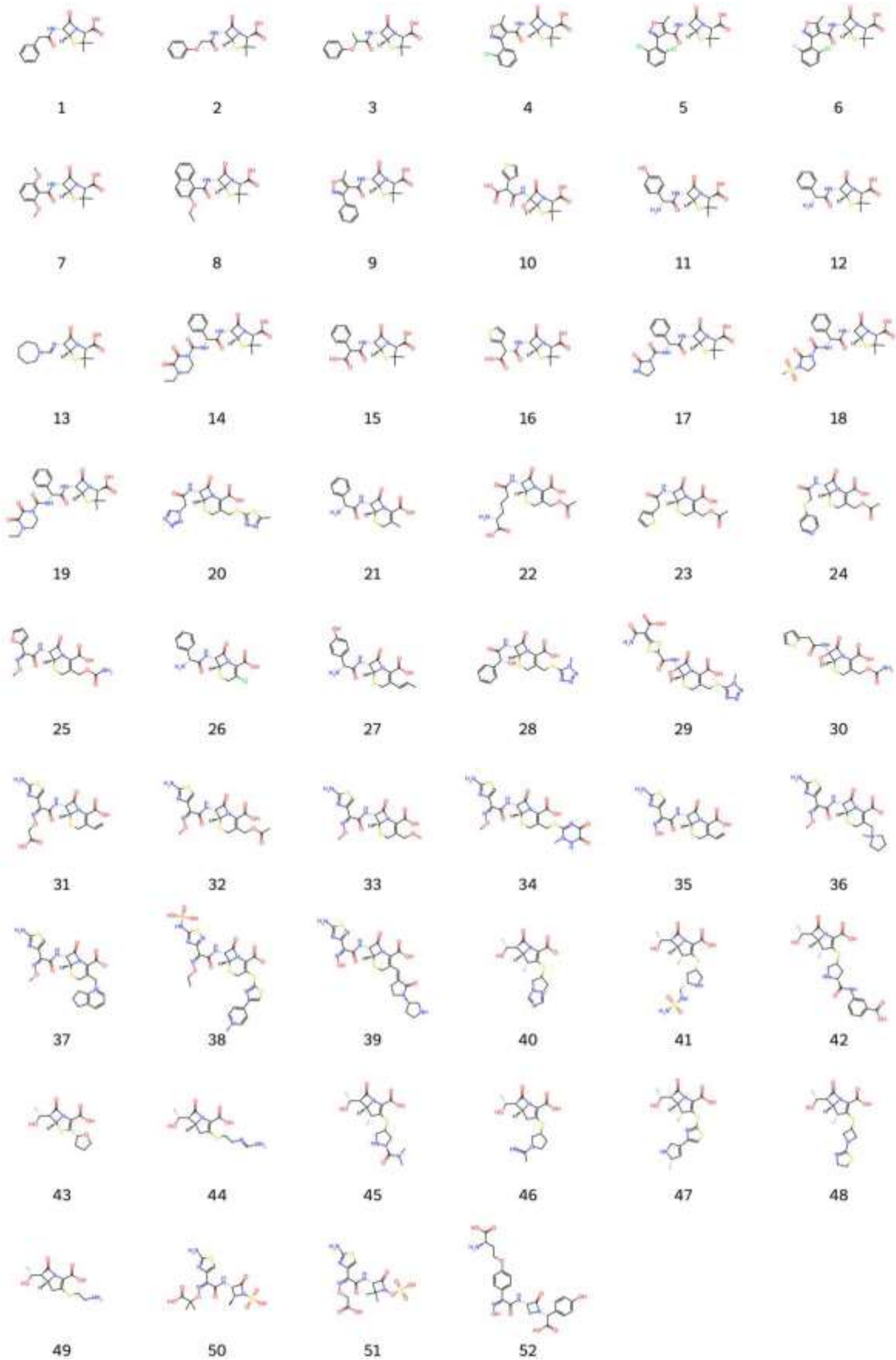

Figure A17: Overview of selected beta-lactam antibiotics

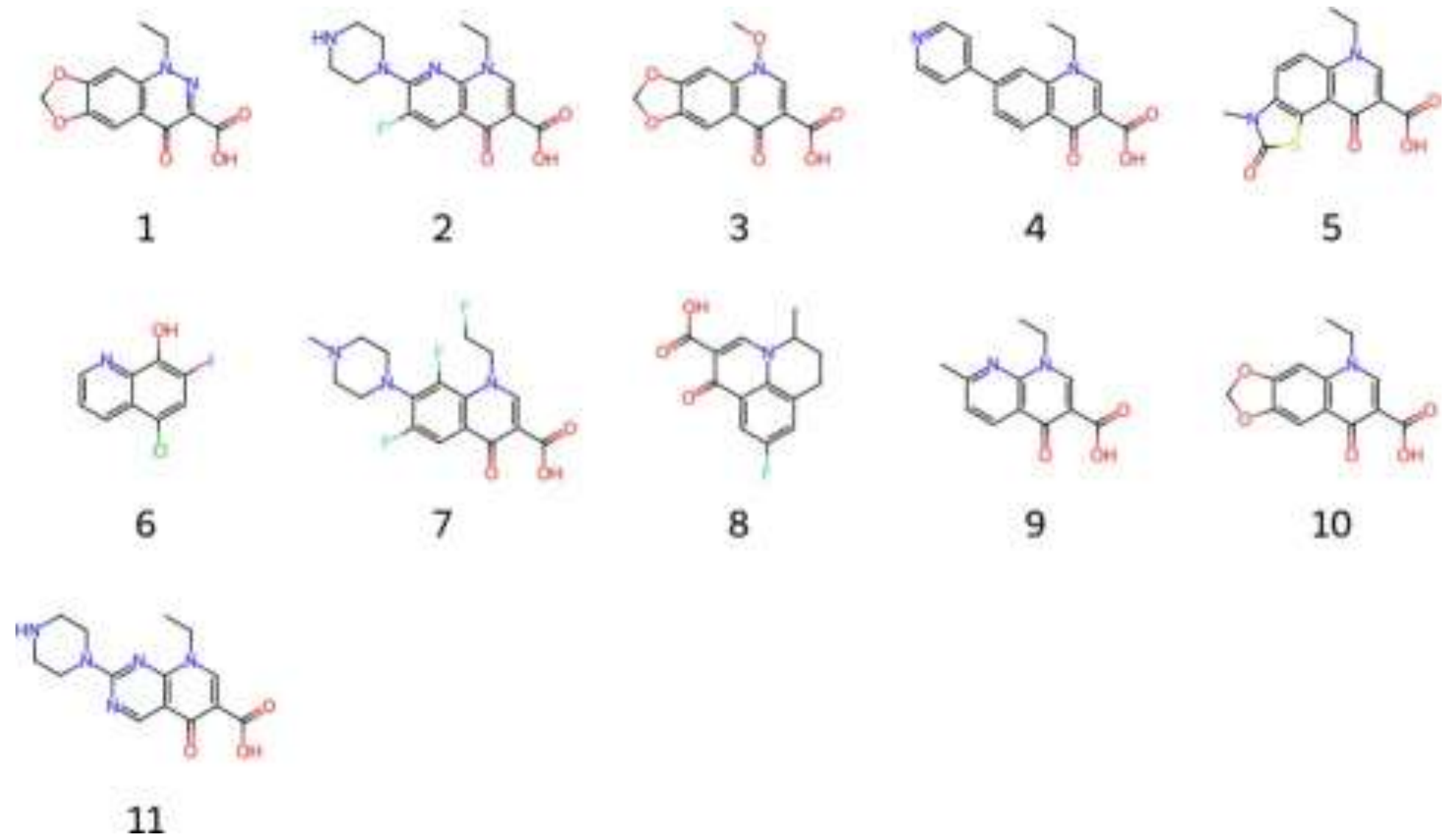

Figure A18: Overview of selected gyrase-inhibiting antibiotics

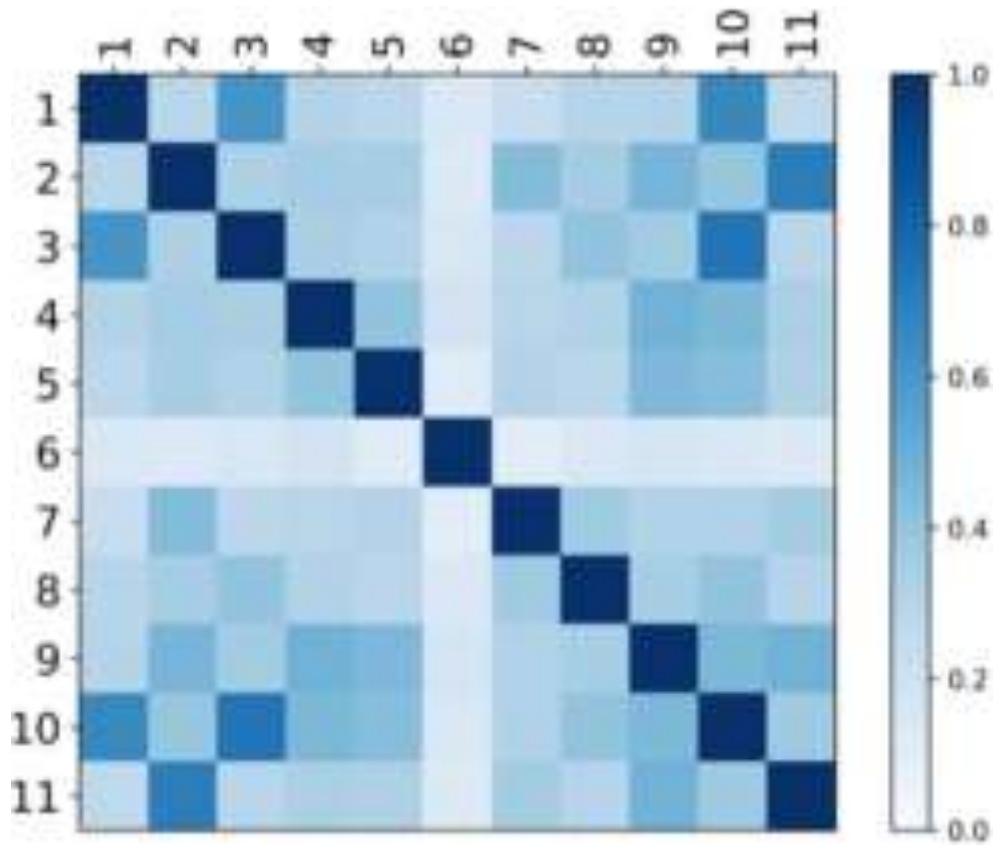

Figure A19: Pairwise similarity relationship between compounds of the gyrase-inhibiting antibiotics set

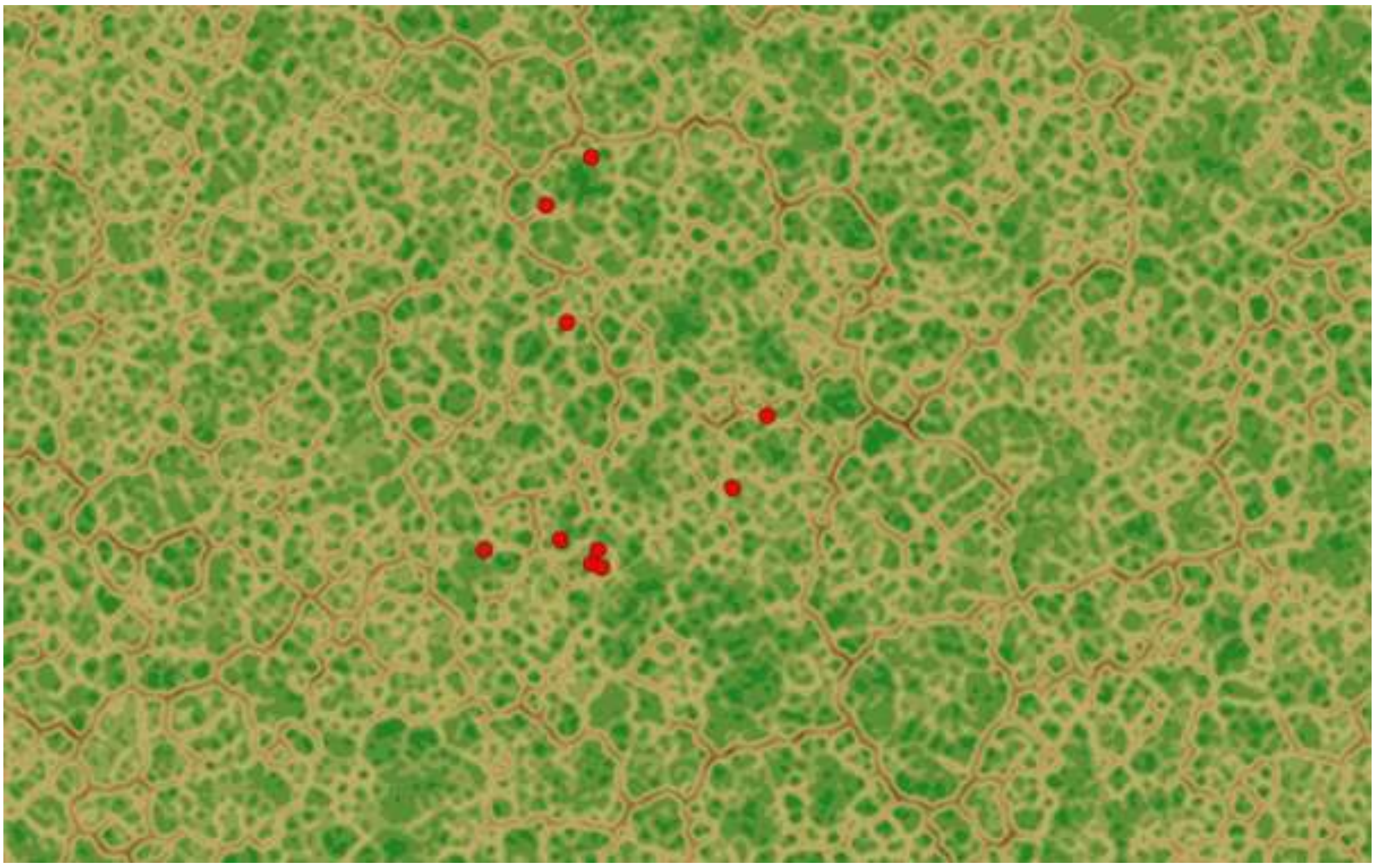

Figure A20: Overview of the map area occupied by gyrase-inhibiting antibiotics

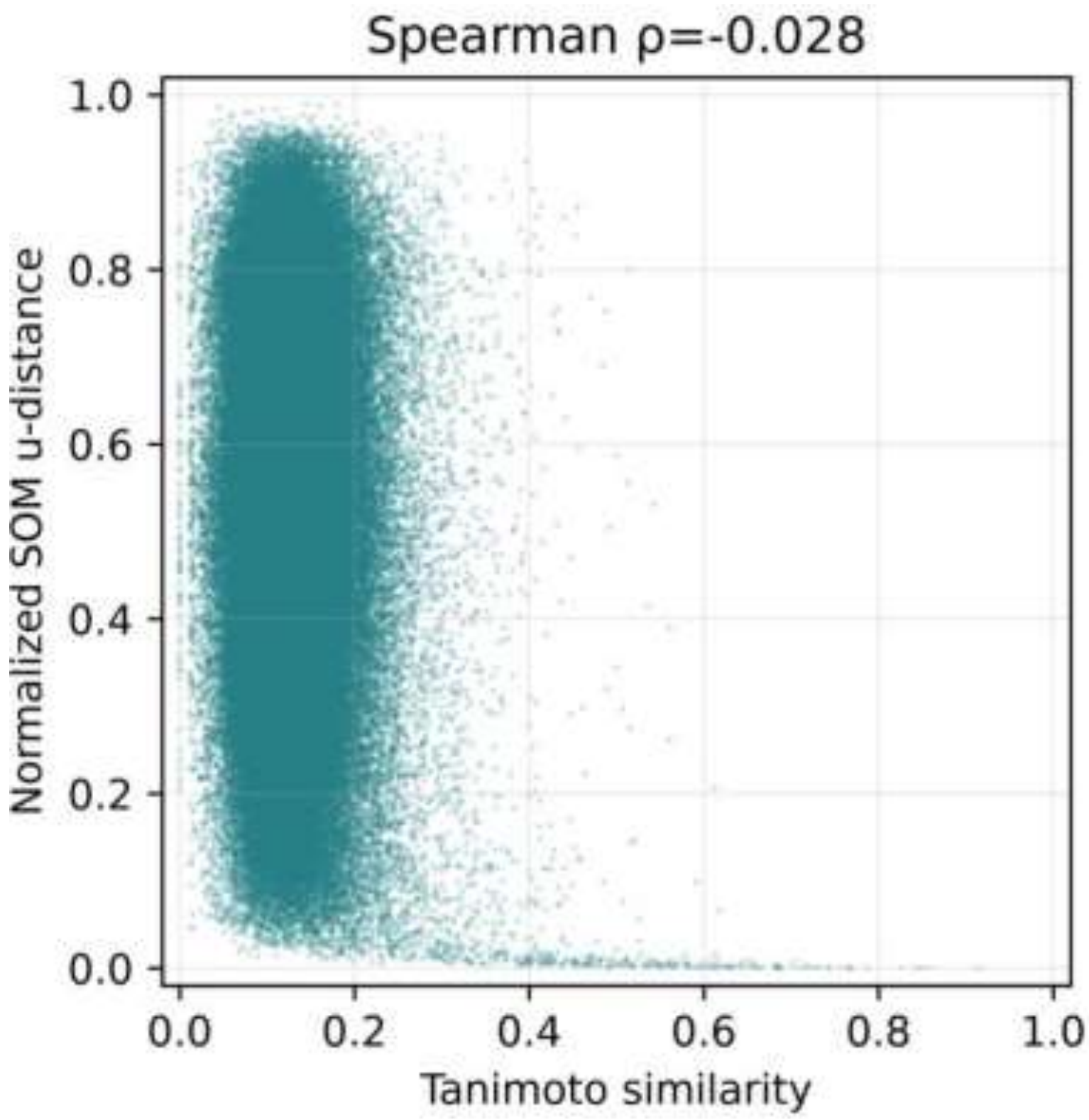

Figure A21: Shepard diagram with Pearson correlation of the ChEMBL33 ESOM. Embedding distance was normalized using min-max-scaling.

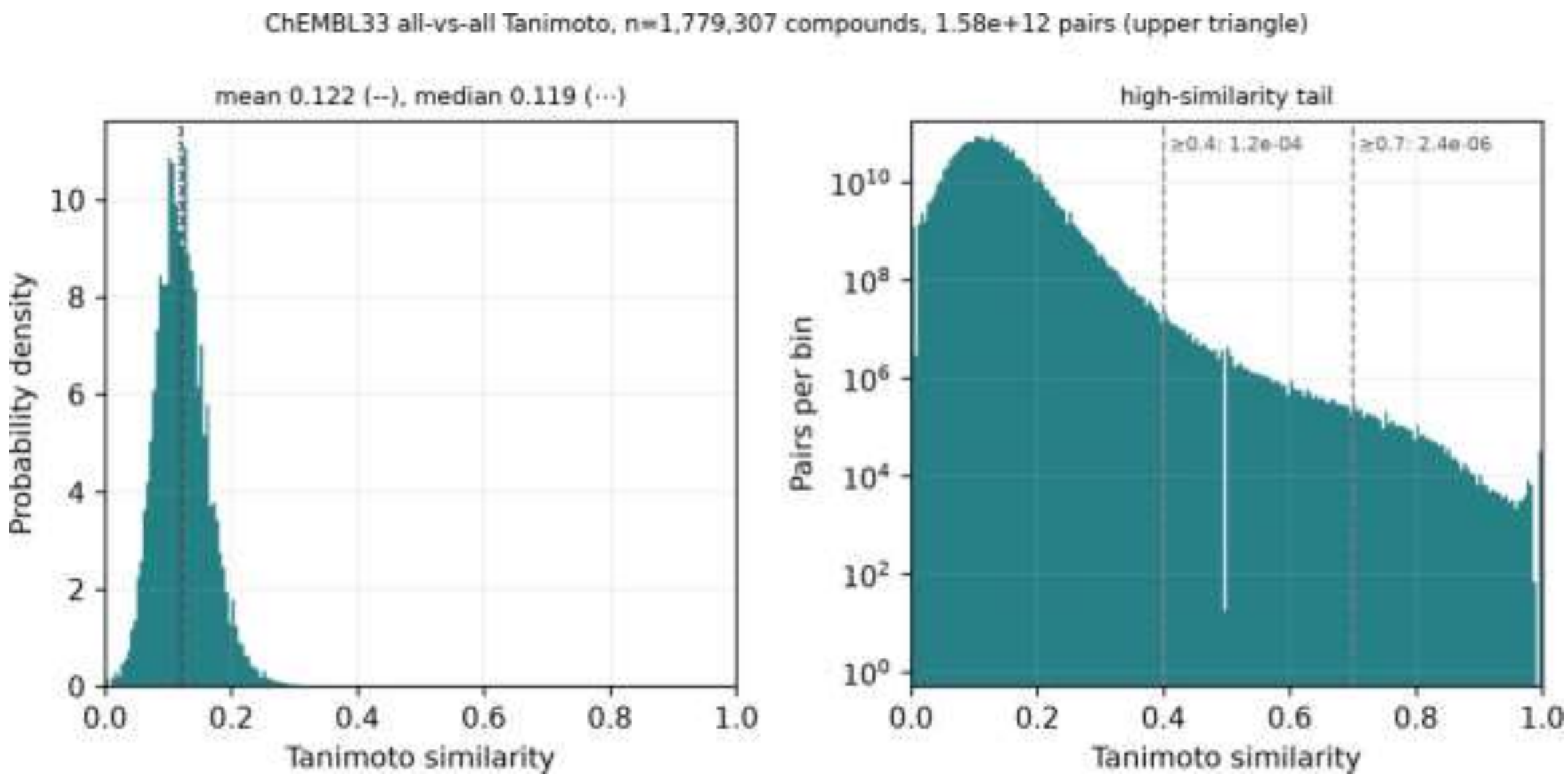

Figure A22: Histograms of the Tanimoto similarity distribution of all compound pairs within the ChEMBL33 dataset. Left panel shows the probability density of a pair distance falling into the specific bin as $(n_{pairs\_in\_bin}/n_{total\_pairs}) * bin\_width$ to remove the influence of the number of bins from the distribution. Right panel show the absolute number of pairs per bin on a logarithmic scale, to make the high-similarity tail visible.

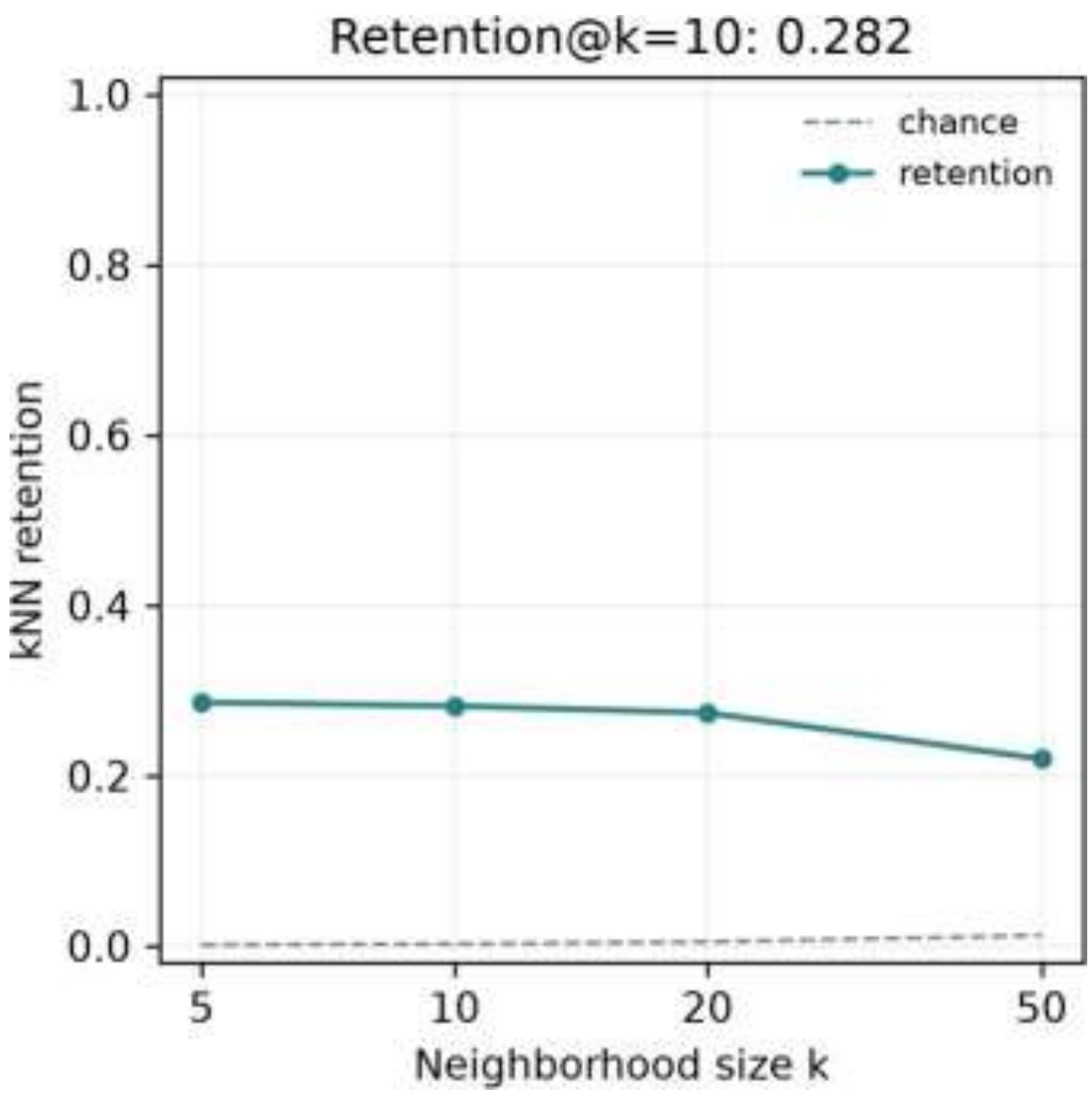

Figure A23: k-Nearest-Neighbor retention for the ChEMBL33 ESOM at different values of k. Dashed line shows the expected value if embeddings are place randomly.

Table A1: Overview of available implementations.

Name	Parallelization	GUI	Cosine Distance
**SimpSOM** [1]	Yes (GPU)	Yes	Yes
**IntraSOM** [2]	Yes (CPU)	U-Matrix, Neuron Map, Toroidal Projection, Component Plots	No
**sparse-som** [3]	Yes (CPU)	No	No
**SuSi** [4]	Yes (CPU)	U-Matrix, Hit map	Yes (Tanimoto)
**Somoclu** [5]	Yes (Cluster + GPU)	No	No
**ESOM Tools** [6]	No		Yes
**MiniSom** [7]	No	Class assignment, Seed maps	No
**sklearn-som**	No	Hit map	No
**som**	No	No	No
**som-pbc**	No	U-Matrix, Seed map, Class assignment	No
**SOMPY** [8]	No	U-Matrix, Hit map	No

Table A2: Software sources

Name	Source
**SimpSOM** [1]	github.com/fcomitani/simpsom
**IntraSOM** [2]	github.com/InTRA-USP/IntraSOM
**sparse-som** [3]	github.com/yoch/sparse-som
**SuSi** [4]	github.com/felixriese/susi
**Somoclu** [5]	github.com/peterwittek/somoclu
**ESOM Tools** [6]	databionic-esom.sourceforge.net
**MiniSom** [7]	github.com/JustGlowing/minisom/
**sklearn-som**	github.com/rileypsmith/sklearn-som
**Som**	github.com/giannisnik/som
**Som-pbc**	github.com/alexarnimueller/som
**SOMPY** [8]	github.com/sevamoo/SOMPY

References

1. fcomitani, Tangherloni A, RichardScottOZ, et al (2023) fcomitani/simpsom: v3.0.0.b

2. Gouvêa RCT de, Gioria R dos S, Marques GR, Carneiro C de C (2023) IntraSOM: A comprehensive Python library for Self-Organizing Maps with hexagonal toroidal maps training and missing data handling. Softw Impacts 17:100570. https://doi.org/https://doi.org/10.1016/j.simpa.2023.100570

3. Melka J, Mariage J-J (2017) Efficient Implementation of Self-Organizing Map for Sparse Input Data. In: Proceedings of the 9th International Joint Conference on Computational Intelligence: IJCCI. SciTePress, Funchal, Madeira, Portugal, pp 54–63

4. Riese FM, Keller S, Hinz S (2020) Supervised and Semi-Supervised Self-Organizing Maps for Regression and Classification Focusing on Hyperspectral Data. Remote Sens 12:. https://doi.org/10.3390/rs12010007

5. Wittek P, Gao SC, Lim IS, Zhao L (2017) somoclu: An Efficient Parallel Library for Self-Organizing Maps. J Stat Softw 78:1–21. https://doi.org/10.18637/jss.v078.i09

6. Ultsch A, Moerchen F (2005) ESOM-Maps: tools for clustering, visualization, and classification with Emergent SOM. Department of Mathematics and Computer Science, University of Marburg, Marburg

7. Vettigli G (2018) MiniSom: minimalistic and NumPy-based implementation of the Self Organizing Map

8. Moosavi V, Packmann S, Vallés I (2014) SOMPY: A Python Library for Self Organizing Map (SOM)
